# Supplementary material for: Bayesian bilevel operator learning with low-rank adaptation for efficient uncertainty quantification of PDE inverse problems
Source: Nat Commun. 2026 Sep 19;17:10979. doi: 10.1038/s41467-026-77768-7 (PMC13683411; doi:10.1038/s41467-026-77768-7)
Supplement: Supplementary file 1 [file 41467_2026_77768_MOESM1_ESM.pdf]

Supplementary Information for: Bayesian Bilevel Operator Learning with Low-Rank Adaptation for Efficient Uncertainty Quantification of PDE Inverse Problems

Ray Zirui Zhang[1*], Christopher E. Miles[2], Xiaohui Xie[3], John S. Lowengrub[2,4*]

[1*]Department of Mathematical Sciences, Worcester Polytechnic Institute, Worcester, 01609, MA, USA.
[2*]Department of Mathematics, University of California, Irvine, Irvine, 92697, CA, USA.
[3]Department of Computer Science, University of California, Irvine, Irvine, 92697, CA, USA.
[4]Department of Biomedical Engineering, University of California, Irvine, Irvine, 92697, CA, USA.

*Corresponding author(s). E-mail(s): rzhang8@wpi.edu; jlowengr@uci.edu;
Contributing authors: chris.miles@uci.edu; xhx@uci.edu;

Supplementary Note 1 BiLO for PDE-Constrained Optimization

For completeness, we briefly review the BiLO framework for solving PDE-constrained optimization problems developed in [1]. We consider the following PDE-constrained optimization problem:

$$\begin{aligned} \min_{\theta} \quad & \|u - \hat{u}\|_2^2 \\ \text{s.t.} \quad & F(\mathcal{D}^k u(\mathbf{x}), ..., \mathcal{D}u(\mathbf{x}), u(\mathbf{x}), \theta) = \mathbf{0} \end{aligned} \tag{A1}$$

where $\hat{u}$ is the observed data.

Using the same definition of the local operator and local operator loss as in the main text, we solve the following bi-level optimization problem:

$$\begin{cases} \theta^* = \arg\min_\theta \mathcal{L}_{\text{dat}}(\theta, W^*(\theta)) \\ W^*(\theta) = \arg\min_W \mathcal{L}_{\text{LO}}(\theta, W) \end{cases} \tag{A2}$$

In the upper level problem, we find the optimal PDE parameters θ by minimizing the data loss $\mathcal{L}_{\text{dat}}(\theta, W) = \|u(\cdot, \theta; W) - \hat{u}\|_2^2$ with respect to θ. In the lower level problem, we train a network to approximate the local operator $u(\mathbf{x}, \theta; W)$ by minimizing the local operator loss with respect to the weights of the neural network.

The bi-level optimization problem (A2) can be solved by simultaneous gradient descent at both levels:

$$\begin{cases} \theta^{k+1} = \theta^k - \alpha_\theta \nabla_\theta \mathcal{L}_{\text{dat}}(\theta^k, W^k) \\ W^{k+1} = W^k - \alpha_W \nabla_W \mathcal{L}_{\text{LO}}(\theta^k, W^k) \end{cases}$$

Theoretically, we show that when the lower-level problem is solved exactly, the gradient of the upper-level loss is exact. When the lower-level problem is solved to a tolerance ϵ, the error between the approximate and exact upper-level gradient is of order ϵ (see Theorems 1 and 2 in [1]).

In [1], the effectiveness of the method is demonstrated on a variety of PDEs, including inferring the diffusion and proliferation rate of Fisher-KPP equation, inferring stochastic rate from particle data (elliptic PDE with singular forcing), inferring the initial condition of a heat equation and inviscid Burgers' equation, inferring the spatially varying diffusion coefficient in a 2D Darcy flow problem, and a Glioblastoma inverse problem using patient data. The method is compared to PINN, Neural Operator, and adjoint methods. Overall, we showed that BiLO is robust to sparse and noisy data, and eliminates the need to balance the residual and the data loss [1].

Supplementary Note 2 Theoretical Results

This note provides theoretical analysis for the B-BiLO method. Section 2.1 establishes the setup and reviews the hypergradient error bound from [1]. Section 2.2 establishes the approximate sampling guarantee: the Wasserstein-1 distance between the θ-marginal invariant distribution of the augmented B-BiLO Markov chain and the ideal posterior (Theorem 4).

2.1 Setup and Hypergradient Error

We first briefly review the theoretical analysis of BiLO on the error of the upper-level gradient, introduced by the inexact minimization of the lower-level problem, which is presented in [1].

Setup

Consider a bounded domain $\Omega \subset \mathbb{R}^d$. The PDE parameter $\theta \in \Theta \subset \mathbb{R}^m$. The PDE operator $\mathcal{F} : H_0^1(\Omega) \times \Theta \to H^{-1}(\Omega)$.

$$\mathcal{F}[u, \theta](\mathbf{x}) := F(\mathcal{D}^k u(\mathbf{x}), ..., \mathcal{D}u(\mathbf{x}), u(\mathbf{x}), \theta) \tag{B3}$$

A PDE can be written as $\mathcal{F}[u, \theta] = \mathbf{0}$. We denote the solution of the PDE for a given θ by u_θ, which satisfies $\mathcal{F}[u_\theta, \theta] = \mathbf{0}$. We define the parameter-to-solution map $\mathcal{G} : \Theta \to H_0^1(\Omega)$ by $\mathcal{G}(\theta) = u_\theta$. The potential energy of the posterior distribution is defined as

$$U(\theta) = \Phi[\mathcal{G}(\theta)] - \log P(\theta),$$

where $\Phi[\cdot]$ is the data-misfit functional (negative log-likelihood up to a constant, acting on the PDE solution) and $P(\theta)$ is the prior distribution of θ. Since $-\log P(\theta)$ is independent of W, the entire approximation error resides in the data-misfit term Φ. In this work, we consider functions of the form $u : \Omega \times \Theta \to \mathbb{R}$, which depend on both the spatial-temporal variable $\mathbf{x}$ and the PDE parameters θ. Such a u induces a map $\mathcal{G}_u : \Theta \to H_0^1(\Omega)$ by $\mathcal{G}_u(\theta) = u(\cdot, \theta)$. If $u(\cdot, \theta)$ is the solution of the PDE for all θ, then $\mathcal{G}_u$ is the parameter-to-solution map [2, 3]. For fixed θ, we call u a local operator at θ, if $\mathcal{F}[\mathcal{G}_u(\theta), \theta] = \mathbf{0}$ and $d_\theta \mathcal{F}[\mathcal{G}_u(\theta), \theta] = \mathbf{0}$, where d_θ is the total derivative with respect to θ.

We consider a neural network parametrization of u, denoted by $u(\mathbf{x}, \theta; W)$, where $W \in \mathbb{R}^n$ are the weights of the neural network. This induces an approximate parameter-to-solution map $\mathcal{G}_W(\theta) = u(\cdot, \theta; W)$. The local operator loss can be defined as

$$\mathcal{L}_{\text{LO}}(\theta, W) = \|\mathcal{F}[\mathcal{G}_W(\theta), \theta]\| + w_{\text{rgrad}} \|\nabla_\theta \mathcal{F}[\mathcal{G}_W(\theta), \theta]\|, \tag{B4}$$

where we called the first term the residual loss and the second term the residual-gradient loss, and w_{rgrad} is a fixed weight. In the theoretical estimates, ϵ denotes this residual-norm tolerance. In the implementation, residual terms are often evaluated as mean-squared losses; stopping a squared loss at tolerance ϵ_{MSE} corresponds to a norm-level tolerance of order $\epsilon_{\text{MSE}}^{1/2}$. The potential energy is then approximated by $U(\theta; W) = \Phi[\mathcal{G}_W(\theta)] - \log P(\theta)$.

We assume that the parametrization is sufficiently accurate, in the sense that for any θ, there exists **ideal optimal weights** $W^*(\theta)$ satisfies the PDE for all θ, that is, the parametrized parameter-to-solution map with the optimal weights is exact: $\mathcal{G}_{W^*} = \mathcal{G}$. Its practical **approximation** is denoted by $\overline{W}$, which is obtained by terminating the optimization once the lower-level loss is within a specified tolerance, $\mathcal{L}_{\text{LO}}(\theta, \bar{W}) \leq \epsilon$. We list the assumptions for the theoretical analysis, which are similar to those in [1].

Assumption 2.1 (Assumptions for Hypergradient Analysis) Consider a parameterized PDE $\mathcal{F}(u, \theta) = 0$ on a bounded domain Ω with $\theta \in \mathbb{R}^m$.

(i) **Inexact Minimization:** The lower-level optimization for the weights $\overline{W}$ terminates when the total local operator loss is within a tolerance ϵ: $\mathcal{L}_{\text{LO}}(\theta, \overline{W}) \leq \epsilon$. Since w_{rgrad} is some fixed weight, without loss of generality, we can assume that both the residual loss and the residual-gradient loss are controlled by ϵ. All estimates below are assumed to hold uniformly over admissible lower-level outputs $\bar{W}$ satisfying $\mathcal{L}_{\text{LO}}(\theta, \bar{W}) \leq \epsilon$.

(ii) **PDE Operator Properties:** The PDE operator $\mathcal{F}[u, \theta]$ is Lipschitz continuous with respect to u and θ. The parameter-to-solution map is stable, that is, if $\mathcal{F}[u, \theta] = \mathbf{0}$ and $\|\mathcal{F}[v, \theta]\| \leq \varepsilon$, then $\|v - u\| \leq C\varepsilon$ for some constant C. The linearized operator at u_θ, denoted $\mathbf{L}_{u_\theta}[\cdot] := \mathcal{F}_u(u_\theta, \theta)[\cdot]$, where $\mathcal{F}_u$ is the partial Frechet derivative of $\mathcal{F}$ with respect to u, is Lipschitz continuous in u and stable, that is, if $\mathbf{L}_{u_\theta}[v] = f$, then $\|v\| \leq C\|f\|$ for some constant C.

(iii) **Smoothness:** The data-misfit functional Φ is Lipschitz continuous. Φ' is bounded.

Assumption 2.2 (Sampling regularity) The ideal potential U^* is continuously differentiable, and U^* and ∇U^* satisfy the Lipschitz or growth bounds used in the Hamiltonian perturbation estimates below. Moreover, either Θ is compact, or the exact and approximate one-step HMC trajectories satisfy the first- and second-moment bounds used in the coupling argument. The augmented B-BiLO chain admits an invariant distribution.

The approximate gradient of the potential energy is given by

$$g_a(\theta) = \nabla_\theta U(\theta; \overline{W})$$

where ∇_θ is the partial derivative with respect to θ, which ignores the implicit dependence of $\bar{W}$ on θ. And the true hypergradient is

$$g_{true}(\theta) = d_\theta U(\theta; W^*(\theta))$$

where d_θ is the total derivative with respect to θ, which accounts for the dependence of $W^*(\theta)$ on θ. Since $-\log P(\theta)$ is independent of W, this difference arises solely from the data-misfit term Φ: $\|g_a - g_{true}\| = \|\nabla_\theta \Phi[\bar{u}] - d_\theta \Phi[u^*]\|$. By Theorem 2 of [1], there exists a constant C_g depending on (C_{stab}, L_Φ, $L_{\Phi'}$) and the Lipschitz/stability constants of $\mathbf{L}_u$ such that

$$\|\nabla_\theta \Phi[\bar{u}] - d_\theta \Phi[u^*]\| \leq C_g\, \epsilon, \qquad \text{hence} \qquad \|g_a(\theta) - g_{true}(\theta)\| \leq C_g\, \epsilon.$$

2.2 Approximate Sampling Guarantee

Perturbation framework.

The implementation carries the auxiliary weights W between proposals, so B-BiLO is Markov on the augmented state space (θ, W), while inference uses only the θ-marginal. Let P_θ^n denote the ideal HMC law after n steps from θ, with $P_\theta = P_\theta^1$. For a carried weight W_{in}, let $\bar{P}_{W_{\text{in}}}(\theta, \cdot)$ be the next-θ law generated by Algorithm 1, including the initial lower-level re-adaptation at θ. For HMC with leapfrog (see Section 4 for a review), the leapfrog step is (1) volume-preserving and (2) time-reversible, meaning that flipping the momentum and running the leapfrog backwards returns to the original state. Together with the Metropolis correction, these two properties imply that the ideal HMC chain targets π^* exactly [4]. For B-BiLO, however, the lower-level problem is solved only to tolerance ϵ, which introduces $O(\epsilon)$ errors in the gradient and in the potential energy. It is not immediate that the key properties of the leapfrog integrator still hold, nor that the θ-marginal invariant distribution of the augmented chain is

close to π^*. Throughout this subsection, we exploit the decomposition $U(\theta; W) = \Phi[\mathcal{G}(\theta; W)] - \log P(\theta)$: since $-\log P(\theta)$ is independent of W, the prior gradient is always evaluated exactly, and *all* approximation error lives in the data-misfit term Φ.

We bound the distance between the θ-marginal invariant distribution $\bar{\pi}$ of the augmented B-BiLO chain and the ideal posterior π^* in terms of the lower-level optimization error ϵ. Our main tool is the Markov-chain perturbation theory of Rudolf and Schweizer [5], which bounds the distance between an ideal and a perturbed chain in terms of the one-step kernel difference. Specifically, assume that the ideal chain contracts in $\mathcal{W}_1$: there exist $C_{\text{erg}} > 0$ and $\rho \in (0, 1)$ such that, for all $\theta, \theta' \in \Theta$ and $n \geq 1$,

$$\mathcal{W}_1(P_\theta^n,\, P_{\theta'}^n) \;\leq\; C_{\text{erg}}\, \rho^n\, \|\theta - \theta'\|. \tag{B5}$$

That is, two copies of the ideal chain started from different points get closer in $\mathcal{W}_1$ at geometric rate ρ. This is known to hold under smoothness and strong log-concavity (or tail-growth) conditions on U^* [6]. If in addition the one-step Wasserstein-1 perturbation is finite,

$$\Delta_{\mathcal{W}_1} := \sup_{\theta \in \Theta,\, W_{\text{in}}} \mathcal{W}_1(P_\theta,\, \bar{P}_{W_{\text{in}}}(\theta, \cdot)) < \infty, \tag{B6}$$

then, provided the augmented B-BiLO chain admits an invariant distribution whose θ-marginal $\bar{\pi}$ satisfies $\mathcal{W}_1(\pi^*, \bar{\pi}) < \infty$ (e.g., Θ compact, or both distributions have finite first moment), the Wasserstein perturbation bound of Rudolf and Schweizer [5] (with the trivial Lyapunov function $V \equiv 1$) yields $\mathcal{W}_1(\bar{\pi}, \pi^*) \;\leq\; \frac{C_{\text{erg}}}{1-\rho}\, \Delta_{\mathcal{W}_1}$.

The remainder of this subsection therefore proceeds in three steps: (i) we show that the B-BiLO leapfrog step is *exactly* volume-preserving (Proposition 1); (ii) we show that it is only *approximately* time-reversible, with momentum error $O(\delta t\, \epsilon)$ (Proposition 2); (iii) we then use a synchronous coupling and a discrete Gronwall argument to establish the one-step kernel bound $\Delta_{\mathcal{W}_1} = O(\epsilon\, L\, \delta t)$ (Proposition 3). Combining with the perturbation theorem yields the approximate sampling guarantee in Theorem 4. Throughout the trajectory length $T = L\, \delta t$ is held fixed.

Proposition 1 (Volume Preservation) *For any fixed incoming auxiliary weight W_0, the conditional B-BiLO leapfrog map $\Phi : (\theta_0, r_0) \mapsto (\theta_1, r_1)$ is volume-preserving in the phase space $(\theta, r) \in \mathbb{R}^m \times \mathbb{R}^m$, that is, the Jacobian determinant of Φ is 1.*

Proof One B-BiLO leapfrog sub-step maps $(\theta_0, r_0) \mapsto (\theta_1, r_1)$ with the auxiliary variable W updated internally:

$$r_{1/2} = r_0 - \tfrac{\delta t}{2}\, \nabla_\theta U(\theta_0; W_0), \tag{B7}$$

$$\theta_1 = \theta_0 + \delta t\, M^{-1} r_{1/2}, \tag{B8}$$

$$W_1 = \text{LowerLevel}(\theta_1, W_0), \tag{B9}$$

$$r_1 = r_{1/2} - \tfrac{\delta t}{2}\, \nabla_\theta U(\theta_1; W_1). \tag{B10}$$

The weights W are auxiliary computational variables. They are not sampled from the posterior, but they are carried by the implementation and enter the proposal through the gradient evaluations. Volume preservation concerns the Jacobian of Φ with W_0 held fixed.

Sub-step (B7) (momentum half-kick) has Jacobian

$$J_1 = \begin{pmatrix} I & 0 \\ -\frac{\delta t}{2}\nabla^2_{\theta\theta}U(\theta_0; W_0) & I \end{pmatrix}, \qquad \det J_1 = 1.$$

Sub-step (B8) (position full-kick) has Jacobian

$$J_2 = \begin{pmatrix} I & \delta t\, M^{-1} \\ 0 & I \end{pmatrix}, \qquad \det J_2 = 1.$$

For sub-steps (B9)–(B10), after sub-step (B8), θ_1 is fixed. Define $g(\theta) := \nabla_\theta U(\theta; \mathrm{LowerLevel}(\theta, W_0))$. The combined map is $r_1 = r_{1/2} - \frac{\delta t}{2}\, g(\theta_1)$ with θ_1 unchanged, giving Jacobian

$$J_{34} = \begin{pmatrix} I & 0 \\ -\frac{\delta t}{2}\nabla_\theta g(\theta_1) & I \end{pmatrix}, \qquad \det J_{34} = 1.$$

The form of $\nabla_\theta g$ may be complicated (involving derivatives of the lower-level solver), but this is irrelevant: a lower-triangular block matrix with identity diagonal blocks always has unit determinant. The full Jacobian is $J = J_{34}\, J_2\, J_1$, so $\det J = 1$. □

With W_0 held fixed, this holds regardless of the number of lower-level iterations, the specific form of $\mathcal{L}_{\mathrm{LO}}$, or whether the lower-level solver converges exactly. Next, we quantify the error in time-reversibility.

Proposition 2 (Approximate Time Reversibility) *Assume the forward B-BiLO leapfrog step (B7)–(B10) starts from an admissible weight W_0 satisfying $\mathcal{L}_{LO}(\theta_0, W_0) \le \epsilon$. Let (θ', r') be obtained by flipping momentum and then taking one B-BiLO leapfrog step from $(\theta_1, -r_1)$. Under Assumption 2.1,*

$$\theta' = \theta_0, \qquad \|r' - (-r_0)\| \;\le\; C_g\, \delta t\, \epsilon, \tag{B11}$$

where $C_g = C_g(C_{\mathrm{stab}},\, L_{\Phi'})$ is the gradient-error coefficient of Theorem 2 of [1]. That is, the position reverses exactly, and the momentum error is $O(\delta t\, \epsilon)$.

Proof We trace the reverse step starting from $(\theta_1, -r_1)$. The first three sub-steps reverse the forward step exactly; the fourth introduces an error.

Momentum half-kick (reverse S1). Compute

$$r'_{1/2} = -r_1 - \tfrac{\delta t}{2}\, \nabla_\theta U(\theta_1; W_1).$$

By the forward sub-step (B10), $r_1 = r_{1/2} - \frac{\delta t}{2}\nabla_\theta U(\theta_1; W_1)$, so $r'_{1/2} = -(r_{1/2} - \frac{\delta t}{2}\nabla_\theta U(\theta_1; W_1)) - \frac{\delta t}{2}\nabla_\theta U(\theta_1; W_1) = -r_{1/2}$. This recovers $-r_{1/2}$ exactly.

Position update (reverse S2). Compute $\theta' = \theta_1 + \delta t\, M^{-1}(-r_{1/2}) = \theta_1 - \delta t\, M^{-1} r_{1/2}$. By the forward sub-step (B8), $\theta_1 = \theta_0 + \delta t\, M^{-1} r_{1/2}$, so $\theta' = \theta_0$ exactly.

Lower-level solve (reverse S3). Starting from W_1 at the new position θ_0, the solver computes $W'_0 = \mathrm{LowerLevel}(\theta_0, W_1)$. Because the lower-level problem is solved inexactly, $W'_0 \ne W_0$ in general, even though both satisfy $\mathcal{L}_{\mathrm{LO}}(\theta_0, \cdot) \le \epsilon$. This is the sole source of irreversibility. Writing $u_0 = \mathcal{G}(\theta_0; W_0)$, $u'_0 = \mathcal{G}(\theta_0; W'_0)$, $u^*_0 = \mathcal{G}^*(\theta_0)$.

Momentum half-kick (reverse S4). The final half-kick produces $r' = -r_{1/2} - \frac{\delta t}{2}\, \nabla_\theta U(\theta_0; W'_0)$. Exact reversal would require $r' = -r_0 = -r_{1/2} - \frac{\delta t}{2}\nabla_\theta U(\theta_0; W_0)$. The

prior-gradient term $-\nabla_\theta \log P(\theta_0)$ is the same for both W_0 and W_0' and therefore cancels, so the discrepancy comes entirely from the data-misfit term:

$$r' - (-r_0) \;=\; -\tfrac{\delta t}{2}\left(\nabla_\theta \Phi[u_0'] - \nabla_\theta \Phi[u_0]\right). \tag{B12}$$

Bounding the momentum error. By Assumption 2.1(i), both W_0 and W_0' attain tolerance ϵ at θ_0. By PDE stability (Assumption 2.1(ii)), both solutions are $O(\epsilon)$-close to the ideal solution, $\|u_0 - u_0^*\| \le C_{\text{stab}}\,\epsilon$ and $\|u_0' - u_0^*\| \le C_{\text{stab}}\,\epsilon$. Applying Theorem 2 of [1] at θ_0 separately to W_0 and to W_0' yields

$$\|\nabla_\theta \Phi[u_0] - d_\theta \Phi[u_0^*]\| \le C_g\,\epsilon, \qquad \|\nabla_\theta \Phi[u_0'] - d_\theta \Phi[u_0^*]\| \le C_g\,\epsilon, \tag{B13}$$

where $C_g = C_g(C_{\text{stab}}, L_{\Phi'})$ is the gradient-error coefficient: it depends on the PDE stability constant C_{stab} and the Lipschitz constant $L_{\Phi'}$ of the data-misfit's derivative, together with bounds on $\nabla_\theta u$ from Assumption 2.1(iii). Subtracting the two bounds in (B13) (the common $d_\theta \Phi[u_0^*]$ cancels) and applying the triangle inequality gives $\|\nabla_\theta \Phi[u_0'] - \nabla_\theta \Phi[u_0]\| \le 2C_g\,\epsilon$, and substituting into (B12):

$$\|r' - (-r_0)\| \;\le\; \tfrac{\delta t}{2}\cdot 2C_g\,\epsilon \;=\; C_g\,\delta t\,\epsilon.$$

□

Proposition 3 (One-Step Kernel Perturbation Bound) *Under Assumptions 2.1 and 2.2, and for any fixed integration time $T = L\,\delta t$, the one-step Wasserstein-1 perturbation between the ideal and B-BiLO chains satisfies*

$$\Delta_{\mathcal{W}_1} \;=\; \sup_{\theta\in\Theta,\, W_{\text{in}}} \mathcal{W}_1(P_\theta,\, \bar{P}_{W_{\text{in}}}(\theta,\cdot)) \;\le\; C_K\,\epsilon\,L\,\delta t, \tag{B14}$$

where C_K is a constant depending on $(C_{\text{stab}}, L_\Phi, L_{\Phi'}, L_U, \|M^{-1}\|, T)$.

Proof For a fixed carried weight W_{in}, the B-BiLO transition first computes

$$W_0 = \text{LowerLevel}(\theta_0, W_{\text{in}}),$$

so that $\mathcal{L}_{\text{LO}}(\theta_0, W_0) \le \epsilon$. We denote the ideal leapfrog trajectory by $(\theta_i^*, r_i^*)_{i=0}^L$ and the resulting B-BiLO trajectory by $(\bar\theta_i, \bar r_i, W_i)_{i=0}^L$; both start from the same state, $\theta_0^* = \bar\theta_0 = \theta_0$ and $r_0^* = \bar r_0 = r_0$.

Coupling. We couple the two chains by using the *same* momentum draw $r_0 \sim \mathcal{N}(0, M)$ and the same uniform variable $\xi \sim \text{Unif}(0,1)$ for the Metropolis decision. Conditional on (r_0, ξ), both chains are deterministic, so

$$\mathcal{W}_1(P_{\theta_0},\, \bar{P}_{W_{\text{in}}}(\theta_0,\cdot)) \;\le\; \mathbb{E}_{r_0,\xi}\big[\|\theta^*_{\text{next}} - \bar\theta_{\text{next}}\|\big].$$

Trajectory perturbation. Let $e_i^\theta = \bar\theta_i - \theta_i^*$, $e_i^r = \bar r_i - r_i^*$, with $e_0^\theta = e_0^r = 0$. Subtracting the ideal and B-BiLO leapfrog updates,

$$e^r_{i+1/2} = e^r_i - \tfrac{\delta t}{2}\big[\nabla_\theta U(\bar\theta_i; W_i) - d_\theta U^*(\theta_i^*)\big], \tag{B15}$$

$$e^\theta_{i+1} = e^\theta_i + \delta t\, M^{-1}\, e^r_{i+1/2}, \tag{B16}$$

$$e^r_{i+1} = e^r_{i+1/2} - \tfrac{\delta t}{2}\big[\nabla_\theta U(\bar\theta_{i+1}; W_{i+1}) - d_\theta U^*(\theta^*_{i+1})\big]. \tag{B17}$$

We decompose each gradient difference as

$$\nabla_\theta U(\bar\theta_j; W_j) - d_\theta U^*(\theta_j^*) \;=\; \big[\nabla_\theta U(\bar\theta_j; W_j) - d_\theta U^*(\bar\theta_j)\big] \;+\; \big[d_\theta U^*(\bar\theta_j) - d_\theta U^*(\theta_j^*)\big]. \tag{B18}$$

The first bracket is the B-BiLO gradient bias at $\bar\theta_j$: since the prior gradient is evaluated exactly, this error lives purely in the data-misfit term and is bounded by $C_g\,\epsilon$ (same C_g as

in Prop. 2). The second bracket is bounded by $L_U\,\|e_j^\theta\|$ by the Lipschitz continuity of ∇U^*. Thus each bracketed gradient difference in (B15)–(B17) has norm $\le C_g\,\epsilon + L_U\,\|e_j^\theta\|$.

Define $Z_i = \|e_i^\theta\| + \|e_i^r\|$. From (B15)–(B17), the Lipschitz continuity of ∇U^*, and the uniform gradient-error bound,

$$Z_{i+1} \le (1 + C\,\delta t)Z_i + C_g'\epsilon\,\delta t, \tag{B19}$$

where C and C_g' absorb the constants depending on L_U and $\|M^{-1}\|_{\rm op}$. Since $Z_0 = 0$, discrete Gronwall gives

$$Z_L \le C_g'\epsilon\,T\,e^{CT}, \qquad T = L\delta t. \tag{B20}$$

Thus, for fixed trajectory length T,

$$\|\bar\theta_L - \theta_L^*\| + \|\bar r_L - r_L^*\| \le C_{\rm tr}\epsilon T. \tag{B21}$$

In particular,

$$\|\bar r_L - r_L^*\| \le C_{\rm tr}\epsilon T, \tag{B22}$$

uniformly in δt for fixed T.

Acceptance-probability perturbation. The Metropolis acceptance probability for a proposal (θ_L, r_L) starting from (θ_0, r_0) is

$$\alpha \;=\; \min\bigl(1,\ \exp(-\Delta H)\bigr), \qquad \Delta H = H(\theta_L, r_L) - H(\theta_0, r_0). \tag{B23}$$

Since both chains share (θ_0, r_0), the initial kinetic energies cancel and

$$\Delta\bar H - \Delta H^* \;=\; \bigl[\bar H_L - H_L^*\bigr] \;-\; \bigl[\bar H_0 - H_0^*\bigr].$$

For the initial term, $\bar H_0 - H_0^* = \Phi[\bar u_0] - \Phi[u_0^*]$ (the prior and kinetic terms cancel); by PDE stability and Lipschitz Φ,

$$|\bar H_0 - H_0^*| \;\le\; L_\Phi\,C_{\rm stab}\,\epsilon \;=:\; C_U\,\epsilon.$$

For the final term, by the triangle inequality,

$$|\bar H_L - H_L^*| \;\le\; |U(\bar\theta_L; W_L) - U^*(\bar\theta_L)| \;+\; |U^*(\bar\theta_L) - U^*(\theta_L^*)| \;+\; |K(\bar r_L) - K(r_L^*)| \tag{B24}$$

The first term is bounded by $C_U\,\epsilon$ as the same PDE-stability and Lipschitz-Φ argument applied at $\bar\theta_L$. The second term is bounded by $L_{U^*}\,\|e_L^\theta\|$ by Lipschitz continuity of U^* itself (constant L_{U^*}, distinct from L_U above which is the Lipschitz constant of ∇U^*), which by (B21) is $O(\epsilon\,T)$. For the third term, applying the mean-value theorem to the kinetic energy $K(r) = \frac{1}{2}r^\top M^{-1}r$ gives $|K(\bar r_L) - K(r_L^*)| \le \mu_M\,\|r_{\rm avg}\|\,\|e_L^r\|$ with $r_{\rm avg}$ on the segment between r_L^* and $\bar r_L$; taking expectation over $r_0 \sim \mathcal{N}(0, M)$ gives $\mathbb{E}\,\|r_{\rm avg}\| \le \mathbb{E}\,\|r_L^*\| + \mathbb{E}\,\|e_L^r\| = O(\sqrt{\operatorname{tr} M}) + O(\epsilon\,T)$, so combined with (B22) this term is $O(\epsilon)$ for fixed T. Combining the preceding bounds and taking expectation over the common momentum draw gives

$$\mathbb{E}_{r_0}\bigl|\Delta\bar H - \Delta H^*\bigr| \le C_H\,\epsilon,$$

where C_H depends on the constants in Assumptions 2.1–2.2, the mass matrix, and the fixed trajectory length T. Since $x \mapsto \min(1, e^{-x})$ is 1-Lipschitz,

$$\mathbb{E}_{r_0}\bigl|\bar\alpha - \alpha^*\bigr| \le C_H\,\epsilon. \tag{B25}$$

Case analysis. Under the coupling (same ξ), the chain accepts if $\xi \le \alpha$ and rejects otherwise. Three cases arise:

- **Both accept** ($\xi \le \min(\alpha^*, \bar\alpha)$): the next states are θ_L^* and $\bar\theta_L$, so by (B21) the distance is $\|\theta_L^* - \bar\theta_L\| \le C_{\rm tr}\,\epsilon\,L\,\delta t$; the probability of this event is at most 1.
- **Both reject** ($\xi > \max(\alpha^*, \bar\alpha)$): both chains stay at θ_0, so the distance is 0.

- **Exactly one accepts** ($\min(\alpha^*, \bar{\alpha}) < \xi \leq \max(\alpha^*, \bar{\alpha})$): Let $D(r_0) = \max\{\|\theta_L^* - \theta_0\|, \|\bar{\theta}_L - \theta_0\|\}$. The contribution from this event is $\mathbb{E}[|\bar{\alpha} - \alpha^*| D(r_0)]$. By the Hamiltonian perturbation estimate above and the moment and displacement bounds in Assumption 2.2, this contribution is bounded by $C \epsilon T$.

Combining the three cases,

$$\Delta_{\mathcal{W}_1} \;\leq\; 1 \cdot C_{\mathrm{tr}}\, \epsilon\, L\, \delta t \;+\; 0 \;+\; C\, \epsilon T \;\leq\; C_K\, \epsilon\, L\, \delta t,$$

where C_K depends on $(C_{\mathrm{stab}}, L_\Phi, L_{\Phi'}, L_{U^*}, L_U, \mu_M, T, M)$, as claimed. □

Theorem 4 (Approximate Sampling Guarantee) *Suppose Assumptions 2.1 and 2.2 hold, the integration time $T = L\,\delta t$ is fixed, and the contraction (B5) holds. Let $\widetilde{\pi}$ be an invariant distribution of the augmented B-BiLO chain on (θ, W), and let $\bar{\pi}$ denote its θ-marginal. Then*

$$\mathcal{W}_1(\bar{\pi},\, \pi^*) \;\leq\; C'\, \epsilon\, L\, \delta t, \tag{B26}$$

where C' is a constant depending on the PDE and potential regularity constants $(C_{\mathrm{stab}},\, L_\Phi,\, L_{\Phi'},\, L_{U^*},\, L_U)$, the kinetic parameters $(\|M^{-1}\|,\, M,\, T)$, and the ergodicity constants $(C_{\mathrm{erg}},\, \rho)$ of the ideal chain.

Proof Let $\bar{\pi}$ be the θ-marginal of the invariant augmented law $\widetilde{\pi}$, and let $\bar{\pi}P^n$ denote the distribution obtained by applying n ideal HMC steps to an initial draw from $\bar{\pi}$. Since $\widetilde{\pi}$ is invariant, the θ-marginal after any number of B-BiLO steps from $\widetilde{\pi}$ is again $\bar{\pi}$. Although the θ-process alone need not be Markov, the one-step bound in Proposition 3 is uniform in the incoming weight. Comparing these n B-BiLO steps with n ideal HMC steps, replacing one step at a time, and using this bound together with the contraction (B5), gives

$$\mathcal{W}_1(\bar{\pi}, \bar{\pi}P^n) \leq \Delta_{\mathcal{W}_1} \sum_{j=0}^{n-1} C_{\mathrm{erg}} \rho^j \leq \frac{C_{\mathrm{erg}}}{1-\rho} \Delta_{\mathcal{W}_1}.$$

Since π^* is invariant for the ideal kernel, the same contraction gives

$$\mathcal{W}_1(\bar{\pi}P^n, \pi^*) \leq C_{\mathrm{erg}} \rho^n\, \mathcal{W}_1(\bar{\pi}, \pi^*).$$

Therefore

$$\mathcal{W}_1(\bar{\pi}, \pi^*) \leq \frac{C_{\mathrm{erg}}}{1-\rho} \Delta_{\mathcal{W}_1} + C_{\mathrm{erg}} \rho^n\, \mathcal{W}_1(\bar{\pi}, \pi^*).$$

Letting $n \to \infty$ and then applying Proposition 3 yields

$$\mathcal{W}_1(\bar{\pi}, \pi^*) \;\leq\; \frac{C_{\mathrm{erg}}}{1-\rho}\, \Delta_{\mathcal{W}_1} \;\leq\; C'\, \epsilon\, L\, \delta t,$$

where $C' = \frac{C_{\mathrm{erg}}}{1-\rho} C_K$ absorbs the constant C_K of Proposition 3. □

Supplementary Note 3 Difference from BPINN

We briefly review the Bayesian PINN (BPINN) framework [7] and highlight the differences with BiLO.

Review of BPINN

Within the BPINN framework, the solution of the PDE is represented by a Bayesian neural network $u_{\text{PINN}}(\mathbf{x}, W)$, where W denotes the weights of the neural network that are taken to be random variables. Note that θ is not an input to the neural network. For the Bayesian Neural Network, the prior distribution of the weights, $P(W)$, is usually assumed to be an i.i.d. Normal distribution.

The likelihood of the observation data $D_u = \{(\mathbf{x}_u^{(i)}, \hat{u}^{(i)})\}_{i=1}^{N_u}$, where $\hat{u}^{(i)} = u(\mathbf{x}_u^{(i)}) + \eta_d$, $\eta_d \sim \mathcal{N}(0, \sigma_d^2)$, is given by

$$P(D_u|W) = \prod_{i=1}^{N_u} \frac{1}{\sqrt{2\pi\sigma_d^2}} \exp\left(-\frac{1}{2\sigma_d^2} \left| u_{\text{PINN}}(\mathbf{x}_u^{(i)}, W) - \hat{u}^{(i)} \right|^2 \right) \tag{C27}$$

Notice that the likelihood of the observation data D_u only depends on the neural network weights W, and does not depend on the PDE parameters θ as BiLO does.

In BPINN, we denote the PDE as $\mathcal{F}(u, \theta)(\mathbf{x}) = f(\mathbf{x})$, where $\mathcal{F}$ is the differential operator and f is the forcing term. Given noisy measurements of the forcing term $D_f = \{(\mathbf{x}_f^{(i)}, \hat{f}^{(i)})\}_{i=1}^{N_f}$, where $\hat{f}^{(i)} = f(\mathbf{x}_f^{(i)}) + \eta_f$ and $\eta_f \sim \mathcal{N}(0, \sigma_f^2)$, then

$$P(D_f|W, \theta) = \prod_{i=1}^{N_f} \frac{1}{\sqrt{2\pi\sigma_f^2}} \exp\left(-\frac{1}{2\sigma_f^2} \left| \mathcal{F}[u_{\text{PINN}}(\cdot; W), \theta](\mathbf{x}_f^{(i)}) - \hat{f}^{(i)} \right|^2 \right) \tag{C28}$$

Assuming θ and W are independent, the following joint posterior is sampled using HMC.

$$P(W, \theta|D_u, D_f) \propto P(D_u|W) P(D_f|W, \theta) P(W) P(\theta). \tag{C29}$$

Challenges for BPINNs

One of the main differences between BPINN and BiLO lies in the treatment of the neural network weights, W. For PDE inverse problems, the PDE parameters θ are usually low-dimensional. In the BPINN framework, the weights W are treated as random variables that must be sampled from the joint posterior distribution alongside the PDE parameters θ. However, W is high-dimensional, making sampling challenging.

In contrast, the BiLO framework treats the weights W as deterministic variables. For any given parameter θ in the sampling process, the optimal weights $W^*(\theta)$ are found through a direct optimization of the lower-level problem, as defined in the second equation in Eq. (A2). This approach avoids placing a prior on W and more importantly bypasses the challenge of sampling from the high-dimensional and often complex posterior distribution of the network weights.

Modeling uncertainty in the forcing term f can also be nuanced. In a BPINN, the solution u is represented by a Bayesian neural network, and uncertainty originates from the prior distribution placed on the network weights, $P(W)$. Uncertainty over the weights W then propagates through the differential operator $\mathcal{F}$ to induce a distribution on the model's estimate of the forcing term, $f = \mathcal{F}(u(\cdot; W), \theta)$. This "top-down" uncertainty propagation from the model's prior contrasts with cases where it is more

physically meaningful for uncertainty in the forcing term f itself (e.g., from noisy measurements or an explicit prior, $P(f)$) to propagate to the solution u.

Another nuance lies in the interpretation of σ_f and D_f. When D_f represents noisy physical measurements of the forcing term f, and σ_f is the noise level, the likelihood $P(D_f|W,\theta)$ allows one to incorporate the PDE. However, if no such measurements are available, then the physics-informed component of the likelihood vanishes. On the other hand, if there is no noise in D_f, then σ_f is 0, and the likelihood becomes singular and cannot be sampled by most sampling methods. An alternative interpretation is to view D_f and σ_f as user-defined constructs. Similar to the residual loss in PINNs, they serve as a soft PDE constraint. In this case, σ_f plays the role of a penalty parameter: smaller values lead to more accurate solution of the PDE.

Regardless of the interpretation, having a small σ_f is computationally demanding. This challenge stems from the stability requirements of HMC, which dictates that the leapfrog step size, δt, is limited by the inverse square root of the potential energy's maximum curvature [4]. In the BPINN framework, this maximum curvature is proportional to $\lambda_{\max}/\sigma_f^2$, where $\lambda_{\max}$ is the largest eigenvalue of the Hessian of the unscaled residual loss, $\mathcal{L}_{\text{res}}$. This unscaled loss is often ill-conditioned, with $\lambda_{\max}$ values known to exceed 10^3 [8]. Consequently, applying the HMC stability limit imposes a scaling law on the step size, forcing δt to be of order $\mathcal{O}(\sigma_f/\sqrt{\lambda_{\max}})$. In contrast, the BiLO framework does not require sampling the weights W, and thus avoids the stability limit imposed by the Hessian of the residual loss. This allows BiLO to use larger step sizes, leading more distant proposals and more efficient sampling.

Supplementary Note 4 Review of Markov Chain Monte Carlo (MCMC) Methods

In this section, we provide a quick overview of the sampling methods that appeared in this work, including the Metropolis-Hastings (MH) algorithm and the Hamiltonian Monte Carlo (HMC). These methods are Markov Chain Monte Carlo (MCMC) methods that generate samples from a target distribution by constructing a Markov chain whose stationary distribution is the target distribution.

We present these methods independent of the BiLO framework. This is assuming that we have the parameter-to-solution map $\theta \mapsto u(\cdot,\theta)$, and we can compute the potential energy $U(\theta)$ and the gradient $\nabla_\theta U(\theta)$ accurately. With this assumption, in this section, we temporarily drop the dependence of the potential energy U on the neural network weights W.

Metropolis-Hastings (MH) Algorithm

The Metropolis-Hastings (MH) algorithm [9] is a classical and simple MCMC method. It requires a proposal distribution $Q(\theta'|\theta)$ to propose new samples θ' given the current sample θ. The proposal is then accepted or rejected based on the changes in the potential energy. Algorithm 1, given below, summarizes the MH algorithm for sampling from the potential energy $U(\theta)$. For simple proposal distributions, such as Gaussian or uniform distributions, MH is easy to implement and does not require computing the

gradient of the potential energy. However, it can be inefficient as the acceptance rate may be low [4]. In this work, the MH algorithm is coupled with an accurate numerical PDE solver, and serves primarily as a reference method to assess the accuracy of alternative sampling approaches.

Algorithm 1 Metropolis-Hastings (MH) Algorithm

Require: Initial state $\theta^{(0)}$, proposal distribution $Q(\theta'|\theta)$, number of iterations N

for $k = 1, 2, \ldots, N$ **do**
  Sample $\theta' \sim Q(\cdot|\theta^{(k-1)})$
  Compute acceptance probability

$$\alpha = \min\left(1, \exp\left[U(\theta^{(k-1)}) - U(\theta')\right] \cdot \frac{Q(\theta^{(k-1)}|\theta')}{Q(\theta'|\theta^{(k-1)})}\right)$$

  Sample $u \sim U[0, 1]$
  if $u < \alpha$ **then**
    Accept the proposal: $\theta^{(k)} \leftarrow \theta'$
  else
    Reject the proposal: $\theta^{(k)} \leftarrow \theta^{(k-1)}$
  end if
end for

Hamiltonian Monte Carlo (HMC)

In HMC, an auxiliary momentum variable ρ is introduced, and the Hamiltonian is defined as

$$H(\theta, \rho) = U(\theta) + K(\rho) = -\log P(\theta|D) + \frac{1}{2}\rho^T M^{-1}\rho, \tag{D30}$$

where M is the mass matrix, which is symmetric and positive definite. M can be user-defined or adaptively learned in a warmup phase. HMC samples from the joint distribution $P(\theta, \rho) \propto \exp(-H(\theta, \rho))$ from the Hamiltonian dynamics.

$$\frac{d\theta}{dt} = M^{-1}\rho, \quad \frac{d\rho}{dt} = -\nabla_\theta U(\theta). \tag{D31}$$

While numerous HMC variants exist, our work employs a standard implementation using the leapfrog integrator with a Metropolis-Hastings correction step. This can be viewed as an instance of the MH algorithm, where the proposal distribution is defined by the Hamiltonian dynamics. The scheme requires computing the gradient of the potential energy. Using the Hamiltonian dynamics, we can generate distant proposals with high acceptance rates. This property helps mitigate the random-walk behavior common in simpler MCMC methods, making HMC a particularly effective and widely-used sampler for exploring complex target distributions [4].

Leapfrog Integrator

The Hamiltonian dynamics can be solved using numerical integrators. The most commonly used integrator is the leapfrog method, which is a symplectic integrator that preserves the Hamiltonian structure [4]. We sample a momentum variable ρ from a Gaussian distribution $\mathcal{N}(0, M)$, and then simulate the Hamiltonian dynamics for L steps with a fixed time step size δt. For $i = 0, 1, \dots, L-1$:

$$\begin{aligned} \rho_{i+\frac{1}{2}} &= \rho_i - \frac{\delta t}{2}\nabla_\theta U(\theta_i) \\ \theta_{i+1} &= \theta_i + \delta t M^{-1}\rho_{i+\frac{1}{2}} \\ \rho_{i+1} &= \rho_{i+\frac{1}{2}} - \frac{\delta t}{2}\nabla_\theta U(\theta_{i+1}), \end{aligned} \tag{D32}$$

Both δt and L are hyperparameters that are predefined or adaptively tuned during a warm-up phase.

Metropolis-Hastings Step

Solving the Hamiltonian dynamics numerically introduces error, which can be corrected using the Metropolis-Hastings step [9]. Starting from some state (θ_0, ρ_0), we perform L leapfrog steps to arrive at (θ_L, ρ_L). We then perform a Metropolis-Hastings step: we accept the proposal θ_L with probability α, where $\alpha = \min\left(1, \exp\left(H(\theta_0, \rho_0) - H(\theta_L, \rho_L)\right)\right)$ If the proposal is accepted, we set $\theta^{(k)} = \theta_L$; otherwise, we set $\theta^{(k)} = \theta^{(k-1)}$. The momentum variable ρ is discarded, and is resampled at the start of the next leapfrog step.

HMC Algorithm

We summarize the HMC algorithm in Algorithm 2. $\theta^{(k)}$ denotes the k-th sample, while $\theta_i^{(k)}$ denotes the PDE parameters at the i-th step of the leapfrog integrator for the k-th proposal. We note that sampling remains an active area of research. For example,

Algorithm 2 Hamiltonian Monte-Carlo with Leapfrog Integrator

Require: Initial states for $\theta^{(0)}$ and time step size δt

```
for k = 0, 1, 2, ..., N do
    Sample r ~ N(0, M)
    (θ_0, r_0) ← (θ^(k), r)
    for i = 0, 1, ..., L − 1 do
        r_{i+1/2} ← r_i − (δt/2) ∇_θ U(θ_i)
        θ_{i+1} ← θ_i + δt M^{−1} r_{i+1/2}
        r_{i+1} ← r_{i+1/2} − (δt/2) ∇_θ U(θ_{i+1})
    end for
    α ← min(1, exp(H(θ_0, r_0) − H(θ_L, r_L)))
    With probability α, set θ^(k) ← θ_L^(k); otherwise, set θ^(k) ← θ^(k−1)
end for
```

many hyperparameters in HMC can be made adaptive. Further, there have been many advancements over HMC, such as the No-U-Turn Sampler (NUTS) [10], higher order integrator [11], or stochastic HMC [12] and we defer the use of such methods for future work. For simplicity and for comparison with [7], we take M as the identity matrix, and we use the leapfrog method with fixed step size δt and length L, which is also one of the most common versions of HMC.

Supplementary Note 5 Additional Results

5.1 Example 1: Nonlinear Poisson Problem

For both B-BILO and BPINN, we use 4-layer fully connected neural network with 128 neurons and tanh activations. The model is trained using the Adam optimizer with AMSGrad, with a learning rate of 10^{-3} for the network weights. For HMC, we fix $L = 10$ and cross validate δt. Residual loss is computed on 51 collocation points, and the noisy data is observed at 6 evenly spaced points in the domain.

Fig 1 shows additional inference results for the nonlinear Poisson problem using B-BiLO and BPINN with different σ_f. Table 1 supplements Fig. 2 in the main text and

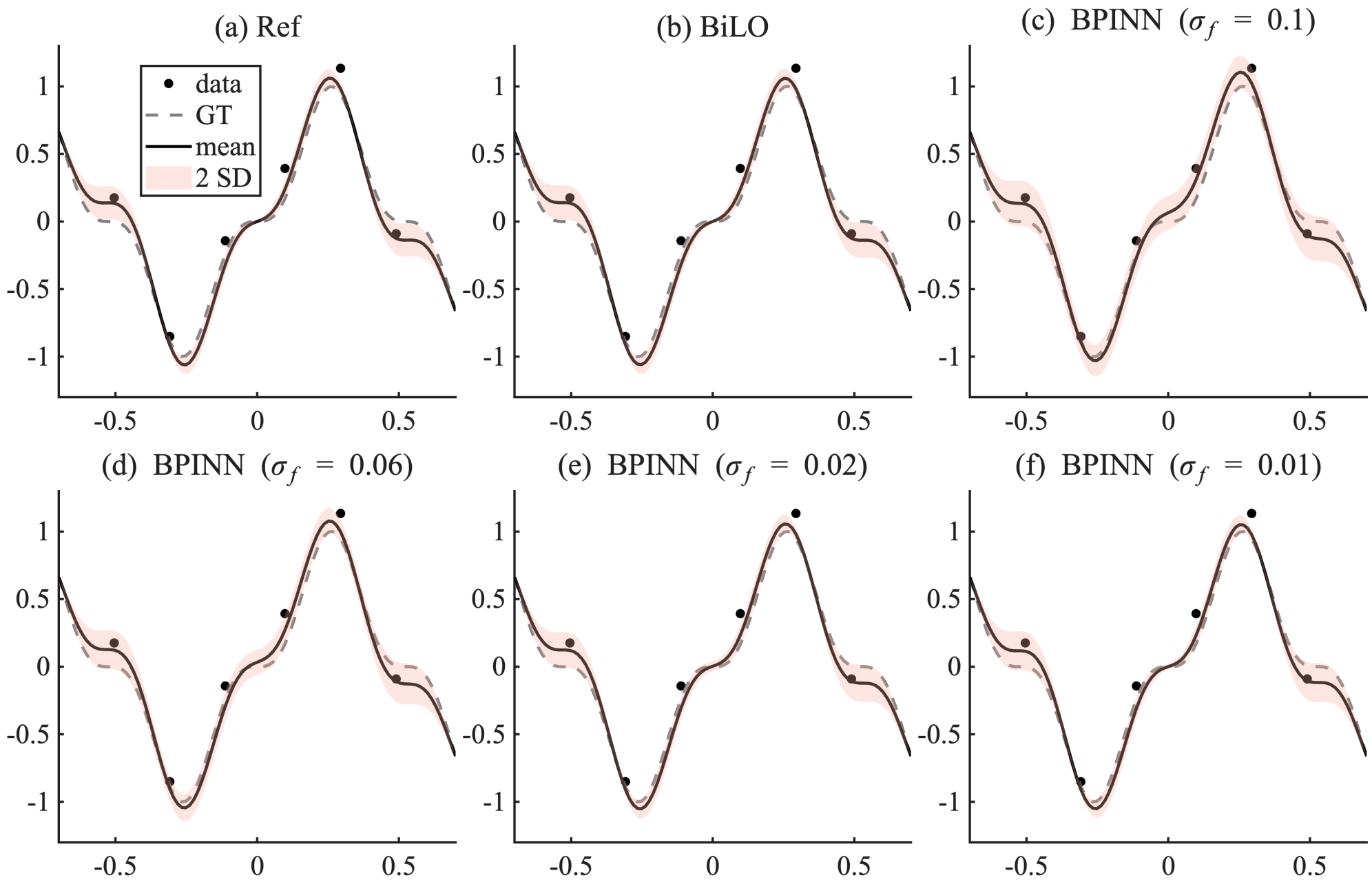

Supplementary Figure 1: Inference results for the nonlinear Poisson problem using B-BiLO and BPINN with different noise levels σ_f. Each panel shows the noisy data, ground-truth (GT) solution, and the mean and standard deviation of the inferred solution u. (a) Reference solution obtained by Metropolis–Hastings (MH) sampling with a numerical PDE solver. (b) B-BiLO. (c–f) BPINN results for various values of σ_f.

shows the mean and standard deviation of the inferred parameter k and the standard deviation of $u(0)$ using different methods.

	mean(k)	SD(k)	SD($u(0)$)
BPINN ($\sigma_f = 0.1$)	0.746	0.043	62.4×10^{-3}
BPINN ($\sigma_f = 0.6$)	0.747	0.033	46.0×10^{-3}
BPINN ($\sigma_f = 0.01$)	0.741	0.034	9.5×10^{-3}
NO (ID)	0.755	0.025	0.6×10^{-3}
NO (OOD)	0.741	0.034	5.5×10^{-3}
BiLO	0.753	0.026	3.0×10^{-3}
Reference	0.754	0.025	5.0×10^{-15}

Supplementary Table 1: Comparison of the mean and standard deviation of k and the standard deviation of $u(0)$ using different methods,

The posterior standard deviation of k is about 0.025, so the ground truth $k = 0.7$ lies within approximately 2 standard deviations of the posterior mean. The discrepancy between the posterior mean (≈0.75) and the ground truth ($k = 0.7$) is attributable to the particular noise realization, not to model mismatch or solver approximation error. In particular: The reference solution, which is obtained by Metropolis–Hastings sampling with a highly accurate numerical solver (finite difference on a fine grid), also yields a posterior mean of approximately 0.754 (see Table 1). This confirms that the shift is not caused by the B-BiLO solver or neural network approximation. The noisy data are generated by adding i.i.d. Gaussian noise to the true solution at only 6 observation points. With such sparse and noisy observations, a single noise draw can noticeably shift the posterior mean away from the ground truth. All methods (B-BiLO, BPINN, and reference) exhibit a similar shift, further supporting that the observed discrepancy is data-driven rather than method-driven.

In addition, we compute the Gelman-Rubin $\hat{R}$ diagnostic for the inferred parameter k using 4 chains (by choosing different random seeds) for B-BiLO. The $\hat{R}$ values are plotted in log scale in Fig. 2. The $\hat{R}$ values are close to 1, indicating good convergence of the sampler.

We also compare against a pretrained neural-operator surrogate based on DeepONet. The surrogate is trained on 101 evenly spaced values of $k \in [0.5, 1]$ and 101 evenly spaced spatial points $x \in [0, 1]$, with an 80/20 train-test split. For the ID setting, the surrogate is pretrained on the full parameter range $k \in [0.5, 1]$, which contains the high-posterior region of this example. For the OOD setting, we intentionally restrict pretraining to $k \in [0.5, 0.7]$ so that the online inference stage queries the surrogate outside its offline training distribution. This setup is deliberately artificial, but it isolates the effect of distribution shift between offline surrogate construction and online Bayesian inference.

The DeepONet uses branch and trunk networks of depth 4 and width 128, for a total of 99,585 trainable parameters. The test mean-squared prediction error is 5.369×10^{-5} for the ID model and 7.9×10^{-5} for the OOD model. As shown in

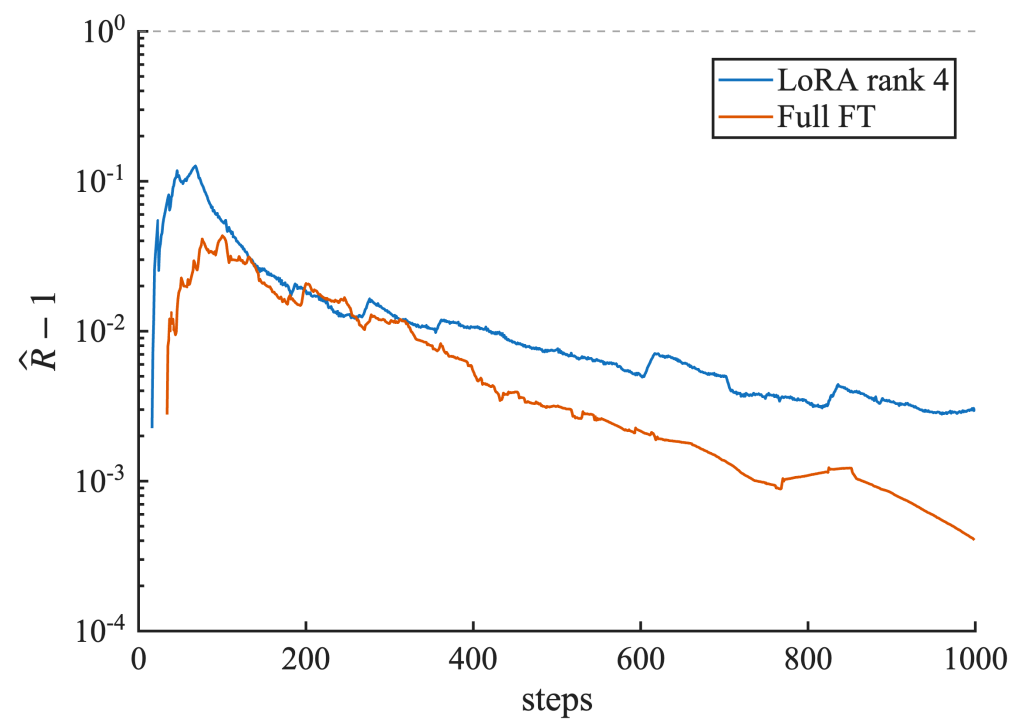

Supplementary Figure 2: Gelman-Rubin $\hat{R}$ diagnostic for the nonlinear Poisson problem. The $\hat{R}$ values are computed from 4 chains with different random seeds for the inferred parameter k. We plot $\hat{R}-1$ on a log scale so that deviations from the ideal value $\hat{R}=1$ are easier to assess; small values indicate that the between-chain and within-chain variances are nearly identical, which is evidence of good mixing and approximate convergence.

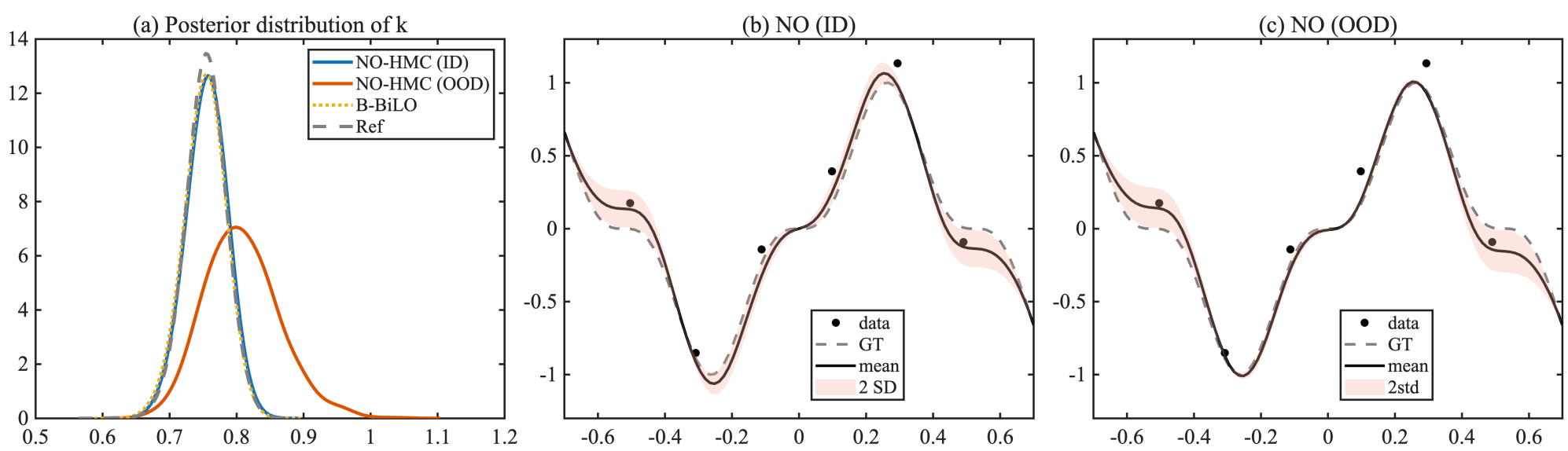

Supplementary Figure 3: Inference results for the nonlinear Poisson problem using a surrogate-based method. We train a DeepONet surrogate for the parameter-to-solution map and then use it to evaluate the potential energy and its gradient within HMC. The surrogate gives accurate posterior estimates in the in-distribution (ID) case, while the out-of-distribution (OOD) case shows a visibly degraded posterior.

Fig. 3 and Table 1, the pretrained surrogate yields a reasonable posterior in the ID case, but the OOD case produces a broader and less accurate distribution for k. At the same time, both surrogates still recover an accurate value for $\mathrm{SD}(u(0))$, because all admissible solutions satisfy the boundary condition at $x=0$. In particular, the OOD result still preserves this feature, which indicates that the surrogate retains some structural generalizability even when its posterior over k deteriorates.

Figure 4 examines the sensitivity of B-BiLO HMC to the lower-level stopping tolerance ϵ and the residual-gradient weight w_{rgrad}. We sweep a 3×3 grid with $\epsilon \in \{10^{-3}, 10^{-4}, 10^{-5}\}$ and $w_{\text{rgrad}} \in \{10^{-3}, 10^{-2}, 10^{-1}\}$, running an independent HMC chain for each pair. In every panel, ϵ is on the horizontal axis on a log scale (decreasing to the right), and the three curves correspond to the three values of w_{rgrad}. Panels

(a)–(c) report chain-averaged lower-level diagnostics on a log y-axis: (a) the residual loss L_{res}; (b) the residual-gradient loss L_{rgrad}; and (c) the *average number of lower-level fine-tuning iterations per HMC proposal*, i.e., the total lower-level step count along the chain divided by the number of upper-level proposals. Panels (d)–(f) report posterior summaries: (d) the posterior mean of k, with a dashed black line marking the reference value from Metropolis–Hastings sampling with a fine-grid numerical solver; (e) the posterior standard deviation of k, again with the MH reference shown as a dashed line; and (f) the posterior standard deviation of $u(0)$, used as a proxy for solution uncertainty—because the PDE imposes $u(0) = 0$ for every k, any spread of $u(0)$ reflects only solver and lower-level approximation error rather than parameter uncertainty.

The dominant trend is that decreasing ϵ from 10^{-3} to 10^{-5} consistently reduces both L_{res} and L_{rgrad}, drives the posterior mean of k closer to the reference value, and decreases the posterior spread in both k and $u(0)$, at the cost of more lower-level iterations. In contrast, at fixed ϵ, varying w_{rgrad} produces comparatively smaller changes in most quantities, although larger w_{rgrad} tends to suppress L_{rgrad} more strongly and can modestly improve posterior concentration. In particular, as w_{rgrad} increases, L_{rgrad} decreases by about an order of magnitude, but L_{res} is largely unaffected. This is because there is no inherent tension between the two losses: a local operator would have these two losses simultaneously 0. Therefore the weight mainly affects the conditioning of the optimization landscape. Overall, larger w_{rgrad} is a more stringent requirement for the lower-level optimization, leading to larger lower-level iteration counts, and can improve posterior concentration; the effect is more subtle than that of ϵ, and for all 3 values of w_{rgrad} tested here, the accuracy of the posterior mean and standard deviation are comparable and the accuracy of the solution uncertainty $\text{SD}(u(0))$ is similar. Overall, the figure indicates that the lower-level tolerance ϵ is the primary driver of both optimization accuracy and posterior quality, while the algorithm is relatively robust to the choice of w_{rgrad}, at least within the tested range.

5.2 Inferring Patient-Specific Tumor Growth Parameters from MRI Data

The setup for the glioblastoma (GBM) tumor growth model is detailed in [13]. The patient brain geometry is obtained by diffeomorphic registration of an anatomical atlas to the patient's MRI, yielding tissue-dependent distributions of white and gray matter, with the diffusion coefficient in white matter assumed to be ten times that in gray matter. We assume the initial tumor cell density to be $u_0(\mathbf{x}) = 0.5 \exp\big(-0.1\|\mathbf{x} - \mathbf{x}_0\|^2\big)$, where $\mathbf{x}_0$ is chosen to be the centroid of the TC segmentation. We assume the predicted segmentations $\mathbf{y}^s(\mathbf{x})$, $s \in \{TC, WT\}$, are related to the tumor cell density $u(\mathbf{x}, 1)$ the tumor cell density u at the nondimensional $t = 1$ via a (soft) thresholding operation: $\mathbf{y}^s(\mathbf{x}) = s(50(u(\mathbf{x}, 1) - u_c^s))$, where $s(x) = 1/(1 + e^{-x})$ is the sigmoid function. To handle the complex brain geometry without meshing, we employ the diffuse-domain method, which embeds the brain into a regular computational domain with a smooth boundary representation. To address the ill-posedness of parameter estimation from single-time MRI data, the PDE is non-dimensionalized following the approach we

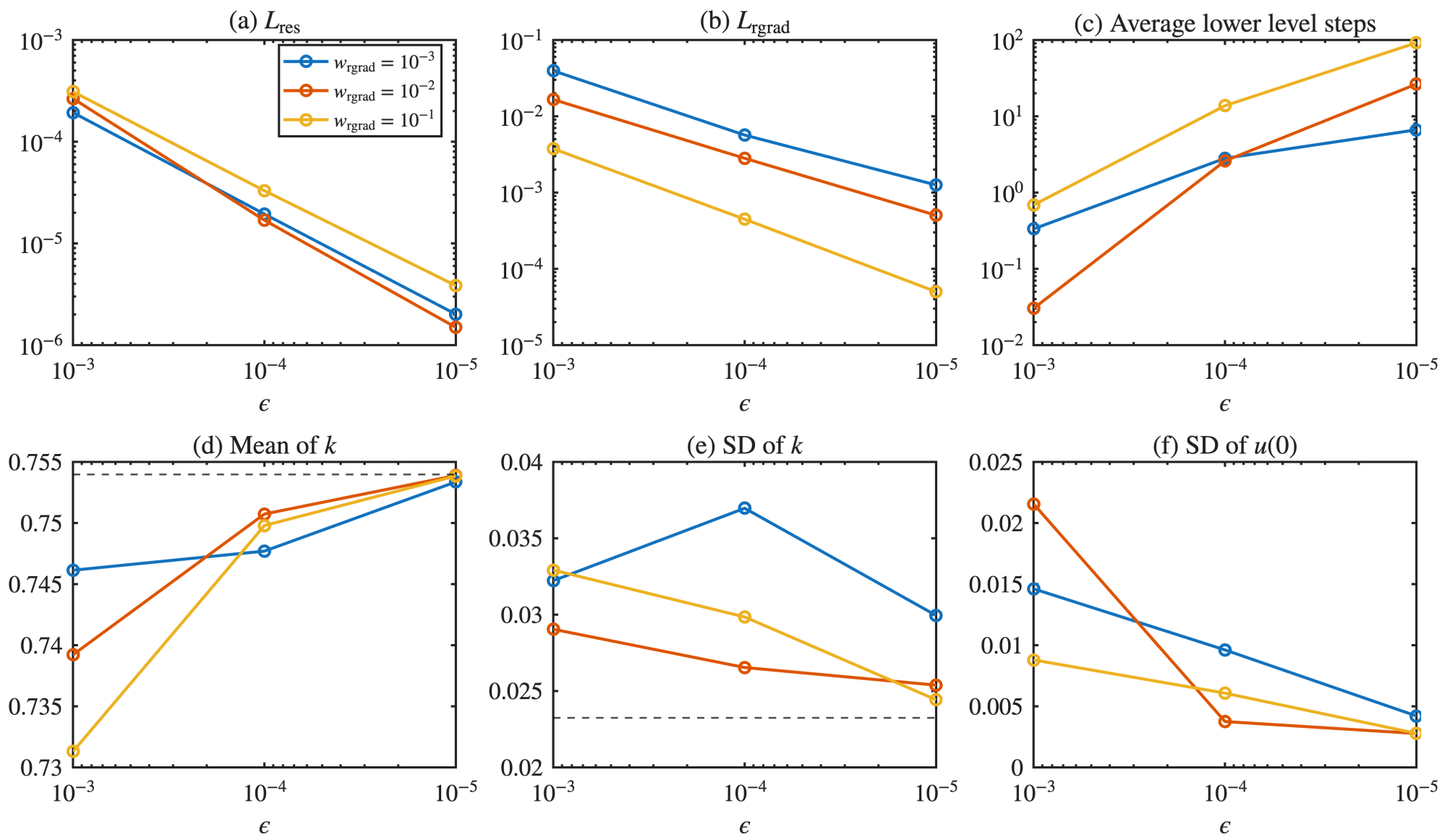

Supplementary Figure 4: Cross-validation results for the nonlinear Poisson problem.

developed in [13]. The Patient-specific characteristic parameters are obtained by ignoring the complex brain geometry, resulting in a radially symmetric PDE in 1D, and selecting the parameters whose predicted tumor radii best match the segmented ones. These characteristic parameters are then used to scale and infer personalized diffusion and proliferation parameters from the MRI segmentations. The inferred parameters enable personalized prediction of tumor cell density and infiltration patterns, which we validate using recurrence data. Compared with the standard uniform-margin clinical target volume, the model-based personalized prediction achieves comparable or improved coverage of the recurrence with reduced irradiation volume, demonstrating its potential for personalized radiotherapy planning [13–15].

Accurately resolving the complex brain geometry and tumor growth dynamics requires a large number of collocation points, which in turn demands substantial GPU memory. Owing to the limited GPU capacity, we adopt a finite difference discretization of the residual and the residual gradient to reduce memory usage [16, 17]. The model is trained using the Adam optimizer with AMSGrad [18, 19] with a learning rate of 10^{-3} for the network weights. The lower-level optimization tolerance is set to 10^{-5}. For the upper-level HMC sampler, we use $L = 10$ leapfrog steps with step size $\delta t = 2 \times 10^{-3}$ and collect $N = 2000$ samples. The solution network is a six-layer modified MLP [20] with 256 neurons per layer, augmented with random Fourier features [21]. This architecture is chosen for improved optimization convergence; however, BiLO is an algorithmic framework and is not restricted to this particular network design.

We first validate our method on synthetic data generated from a known set of parameters $D = 0.894$, $\rho = 0.860$, $u_c^{\text{WT}} = 0.3$, $u_c^{\text{TC}} = 0.5$, $\bar{D} = 0.17$, $\bar{\rho} = 0.31$. The prior $u_c^{\text{TC}} \sim$ Uniform(0.1,0.4), $u_c^{\text{WT}} \sim$ Uniform(0.4,0.8), $D \sim$logNormal(0.1,0.316),

ρ ~logNormal(0.1,0.316). The geometry is a 2D slice of the brain atlas. The results are shown in Fig. 5.

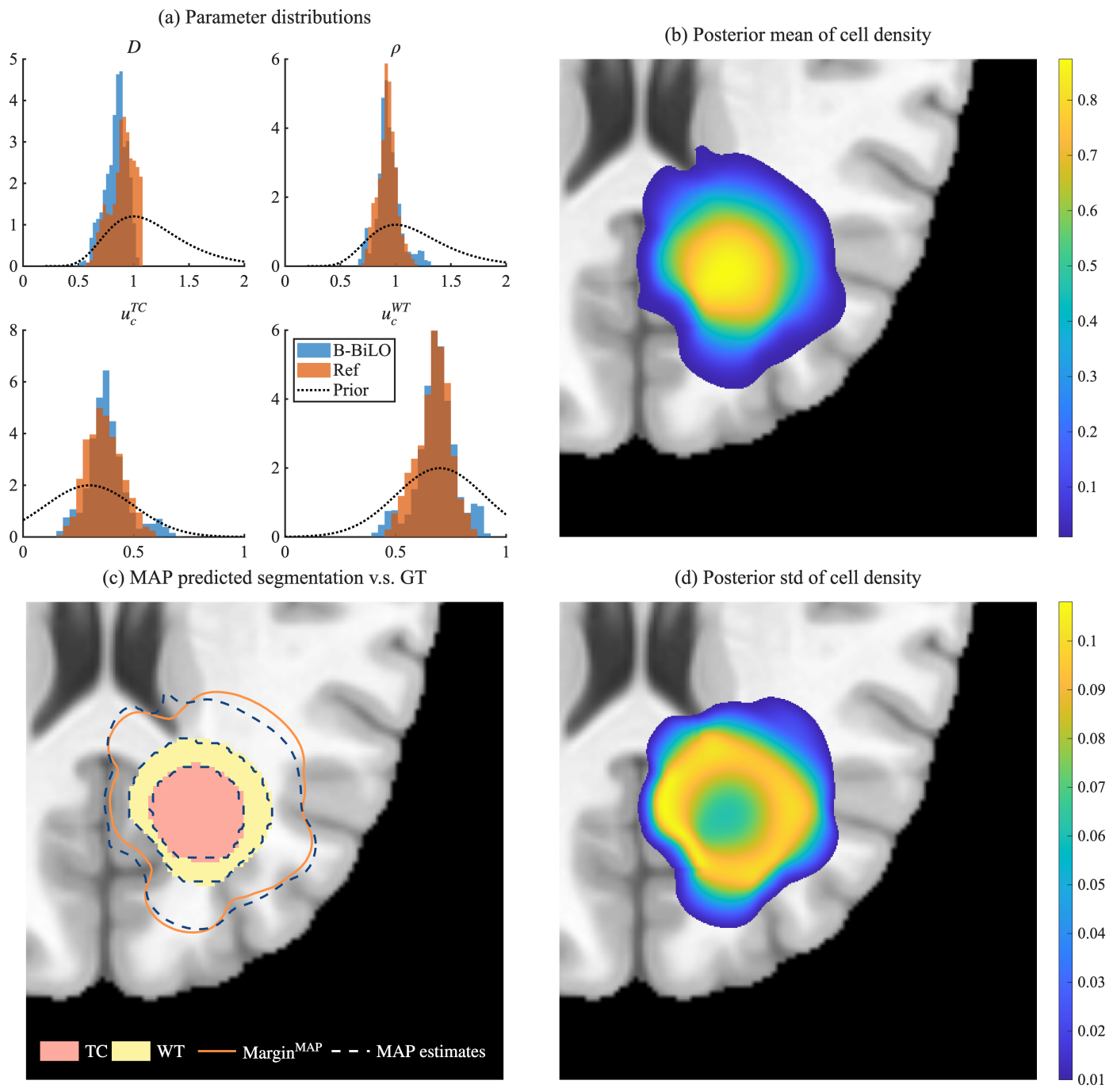

Supplementary Figure 5: BiLO inference on synthetic tumor. (a) The inferred posterior distribution of D, ρ, u_c^{WT}, u_c^{TC} and their prior distributions, using the reference method (MH and numerical solver) and B-BILO rank 4. The legends also indicated the posterior mean and standard deviation. (b) The posterior mean of the tumor cell density. (c) Ground truth WT and TC segmentations (filled) with the infiltration margin (solid orange line), and MAP-predicted segmentations and infiltration margin (dashed lines). (d) The posterior standard deviation of the tumor cell density.

For the patient case shown in the main text, the prior distributions are $u_c^{\mathrm{TC}} \sim \mathrm{Normal}(0.3, 0.1^2)$, $u_c^{\mathrm{WT}} \sim \mathrm{Normal}(0.7, 0.1^2)$, D ~logNormal$(0.01, 0.1)$, ρ ~logNormal$(0.01, 0.1)$.

5.3 Example 3: Inferring Stochastic Rates from Particle Data

The PDE in $\Omega = [0, 1]$ can be written as a elliptic interface problem:

$$\begin{cases} \Delta u - \mu u = 0 \\ u(0) = u(1) = 0 \\ u^+(z) = u^-(z) \\ u_x^+(z) - u_x^-(z) = -\lambda \end{cases}$$

Here, the superscripts + and − indicate the limits from the right and left of the interface point z. While the solution u is continuous across this point, its derivative exhibits a jump discontinuity.

To address the singularity in the forcing term, we employ the cusp-capturing PINN method from [22]. This technique involves learning a function $\bar{u}(x, \phi)$ such that $u(x) = \bar{u}(x, |x - z|)$. This formulation inherently satisfies the continuity condition. The jump condition is incorporated as an additional constraint within $\mathcal{F}$:

$$\partial_\phi \bar{u}(z, 0) = -\lambda.$$

The cusp-capturing PINN is parameterized by $\bar{u}(x, \phi; W)$, and "jump loss" is needed to enforce the jump condition:

$$\mathcal{L}^{PINN}_{\text{jump}}(W) = (\partial_\phi \bar{u}(z, 0; W) + \lambda)^2$$

In our BiLO framework, we parameterize the local operator as $\bar{u}(x, \phi, \theta; W)$, with $\theta = (\lambda, \mu)$. The corresponding "jump loss" is:

$$\mathcal{L}_{\text{jump}}(\theta, W) = (\partial_\phi \bar{u}(z, 0, \theta; W) + \lambda)^2.$$

Additionally, we define a "jump gradient loss" to satisfy the local operator conditions:

$$\mathcal{L}_{\text{jgrad}}(\theta, W) = \|\nabla_\theta \partial_\phi \bar{u}(z, 0, \theta; W)\|^2$$

The lower-level local operator loss is given by $\mathcal{L}_{LO} = \mathcal{L}_{\text{res}} + \mathcal{L}_{\text{jump}} + w_{\text{rgrad}}(\mathcal{L}_{\text{rgrad}} + \mathcal{L}_{\text{jgrad}})$.

For the experiments in this section, we use a 4-layer residual fully connected neural network [23] with width 512 and tanh activations, augmented with random Fourier features [21]. The model is trained using the Adam optimizer [18] with AMSGrad [19], employing a learning rate of 10^{-4} for the network. We collect 5,000 HMC samples with $\delta t = 10^{-2}$ and $L = 50$, yielding ESS of approximately 150. The exact solution to the PDE is given by [24]:

$$u(x) = \frac{\lambda}{\sqrt{\mu}} \operatorname{csch}(\sqrt{\mu}) \sinh(\sqrt{\mu} \min\{x, z\}) \sinh(\sqrt{\mu}(1 - \max\{x, z\}))$$

Due to the relatively large magnitude of the solution, We set $\epsilon = 10$, which results in a relative error of approximately 1% in the infinity norm compared to the exact solution. The local operator loss is evaluated on 101 evenly spaced points in the domain. In this problem, the decay rate μ is typically on the order of 10, while the birth rate λ is on the order of several hundreds, as determined by the biological dynamics of the system. This scale ensures that a sufficient number of particles are present for inference. To improve numerical conditioning during training, we reparameterize $\lambda = 100\bar{\lambda}$ and learn the rescaled parameter $\bar{\lambda}$. We also represent BiLO as $u(x, \theta; W) = m(x, \theta; W)^2 x(1-x)$, where m is the raw output of the MLP. This transformation enforces the boundary

conditions $u(0) = u(1) = 0$ and ensures $u \geq 0$, which is necessary for evaluating $\log u$ in the likelihood.

In Table 2, we show the relative error of the MAP estimate of λ and μ, and the area of 90% HPDR with respect to the reference method.

	Area	λ_{MAP}	μ_{MAP}
Full FT	3.5%	1.8%	3.4%
LoRA rank 4	2.1%	2.0%	4.0%

Supplementary Table 2: relative error of the MAP estimates of λ and μ and the area of 90% HPDR with respect to the reference method

In addition, we compute the Gelman–Rubin $\hat{R}$ diagnostic for the inferred parameters λ and μ using 4 chains (with different random seeds) for B-BiLO with LoRA rank 4. The $\hat{R} - 1$ values are plotted on a log scale in Fig. 6; both parameters drop well below 10^{-2} as sampling progresses, indicating good convergence and mixing for both rates.

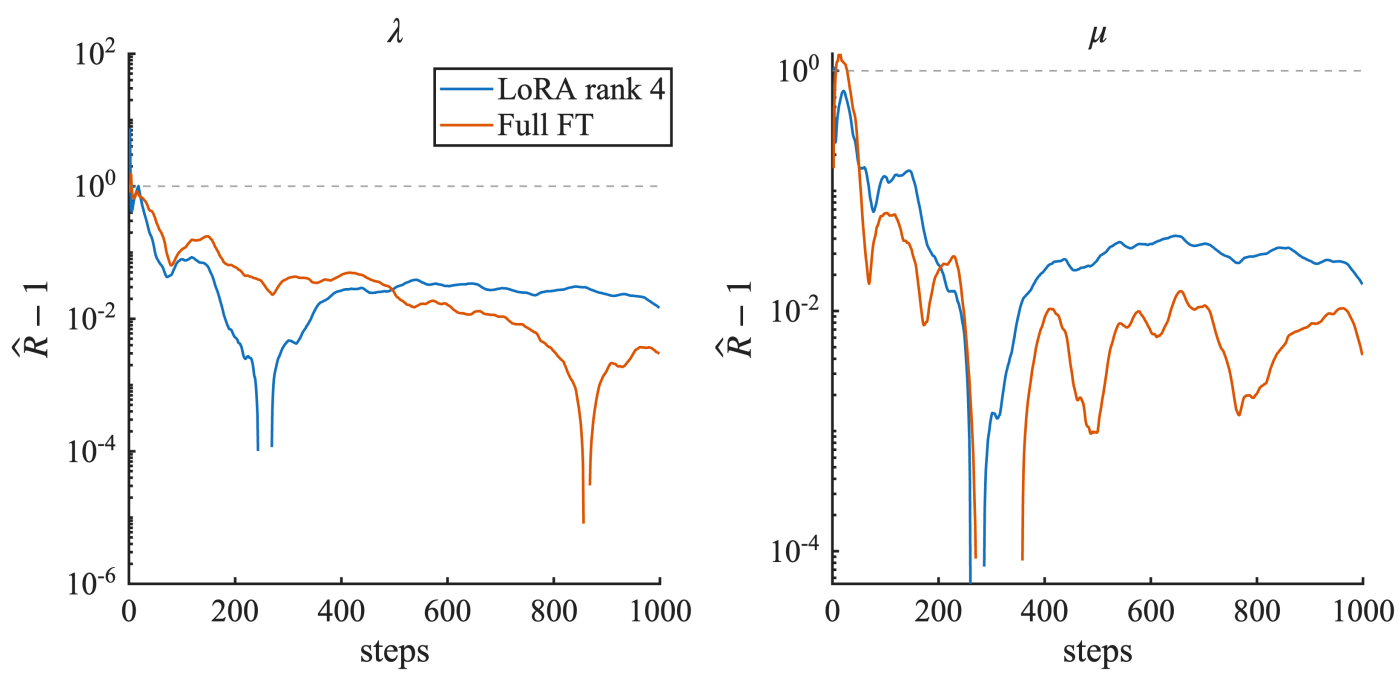

Supplementary Figure 6: Gelman–Rubin $\hat{R}$ diagnostic for the point-process problem. The $\hat{R}-1$ values for the inferred birth rate λ and decay rate μ are computed from 4 chains with different random seeds for B-BiLO with LoRA rank 4 and plotted on a log scale; small values indicate that the between-chain and within-chain variances are nearly identical, which is evidence of good mixing and approximate convergence.

5.4 Darcy Flow Problem

We set the lower-level optimization tolerance to $\epsilon = 0.5$. The neural network is a six-layer residual MLP with 512 neurons per layer [23], augmented with random Fourier features [21]. For the upper-level HMC sampler, we use $L = 100$ leapfrog steps with

step size $\delta t = 10^{-3}$ and collect $N = 5000$ samples. The BiLO model is represented as $u(\mathbf{x}, z; W) = m(\mathbf{x}, z; W)\mathbf{x}_1(1 - \mathbf{x}_1)\mathbf{x}_2(1 - \mathbf{x}_2)$, where m is the raw output of the MLP and z is the auxiliary variable. This form ensures that the Dirichlet boundary conditions are satisfied by construction.

We use a KL expansion to represent the unknown function $D(\mathbf{x})$. Let $g(\mathbf{x}, \theta)$ be the K term KL expansion of a mean zero Gaussian process with covariance kernel $C = (-\Delta + \tau^2)^{-d}$, where $\tau = 3$ and $d = 2$, and $-\Delta$ is the Laplacian on Ω subject to homogeneous Neumann boundary conditions [25, 26]:

$$g(\mathbf{x}, \theta) = \sum_{i=1}^{\sqrt{K}} \sum_{j=1}^{\sqrt{K}} \theta_{ij} \sqrt{\lambda_{ij}} \cos(i\pi x_1) \cos(j\pi x_2), \quad \lambda_{ij} = (\pi^2(i^2 + j^2) + \tau^2)^{-2} \tag{E33}$$

The prior of the KL coefficients is the standard normal distribution. In the example, we assume $D(\mathbf{x}, \theta) = 9s(20g(\mathbf{x}, \theta)) + 3$, where $s(x) = 1/(1 + e^{-x})$ is the sigmoid function. This models a spatially varying diffusion coefficient with low and high diffusivity (3 and 12) in the domain. The initial guess for all simulations is $\theta = \mathbf{0}$.

To assess the scalability of B-BiLO with respect to the dimension of the inferred function, we run the Darcy flow inverse problem with the KL truncation varied as $K = n^2 \in \{16, 64, 256\}$ (i.e., $n \in \{4, 8, 16\}$ in each spatial direction in the expansion of $g(\mathbf{x}, \theta)$). For each K, we draw a fresh set of ground-truth KL coefficients from the corresponding K-dimensional standard normal prior, solve the PDE on a fine grid to obtain u, and generate noisy observations from this solution; the inference is then performed under the same K-truncated KL prior. All other settings—network architecture, leapfrog parameters, observation noise level, and number of HMC iterations—are kept fixed.

Figure 7 shows the ground truth diffusion coefficient $D(\mathbf{x})$, the MAP estimate, the posterior mean, and the posterior standard deviation for each K. The ground truth becomes spatially richer as K grows (more KL modes are excited), and B-BiLO recovers a posterior mean and MAP that closely match the ground truth at each resolution; the posterior standard deviation reflects the spatial distribution of uncertainty in each case.

Figure 8 presents box plots of the leading nine KL coefficients θ_{ij} (small i, j), comparing B-BiLO with full fine-tuning, B-BiLO with LoRA rank 4, and the reference MH sampler with a fine-grid numerical solver. Across all three values of K and all three samplers, the ground-truth coefficients are well captured by the posterior box plots, and the three samplers are mutually consistent. This indicates that the inference in coefficient space is well-calibrated, and that LoRA fine-tuning is essentially as accurate as full fine-tuning.

Table 3 reports (i) the wall-clock per HMC iteration, (ii) the average number of lower-level fine-tuning steps per HMC iteration, (iii) the median effective sample size (ESS) across the K KL coefficients, and (iv) the wall-clock per median ESS. For high-dimensional chains, multivariate ESS estimators that require non-singular sample covariance (e.g., multiESS [27]) are impractical at $K = 256$ with $N = 999$ samples. We therefore compute per-coordinate ESS via Geyer's initial-positive-sequence autocorrelation sum [28] and report the median across coordinates as a robust summary

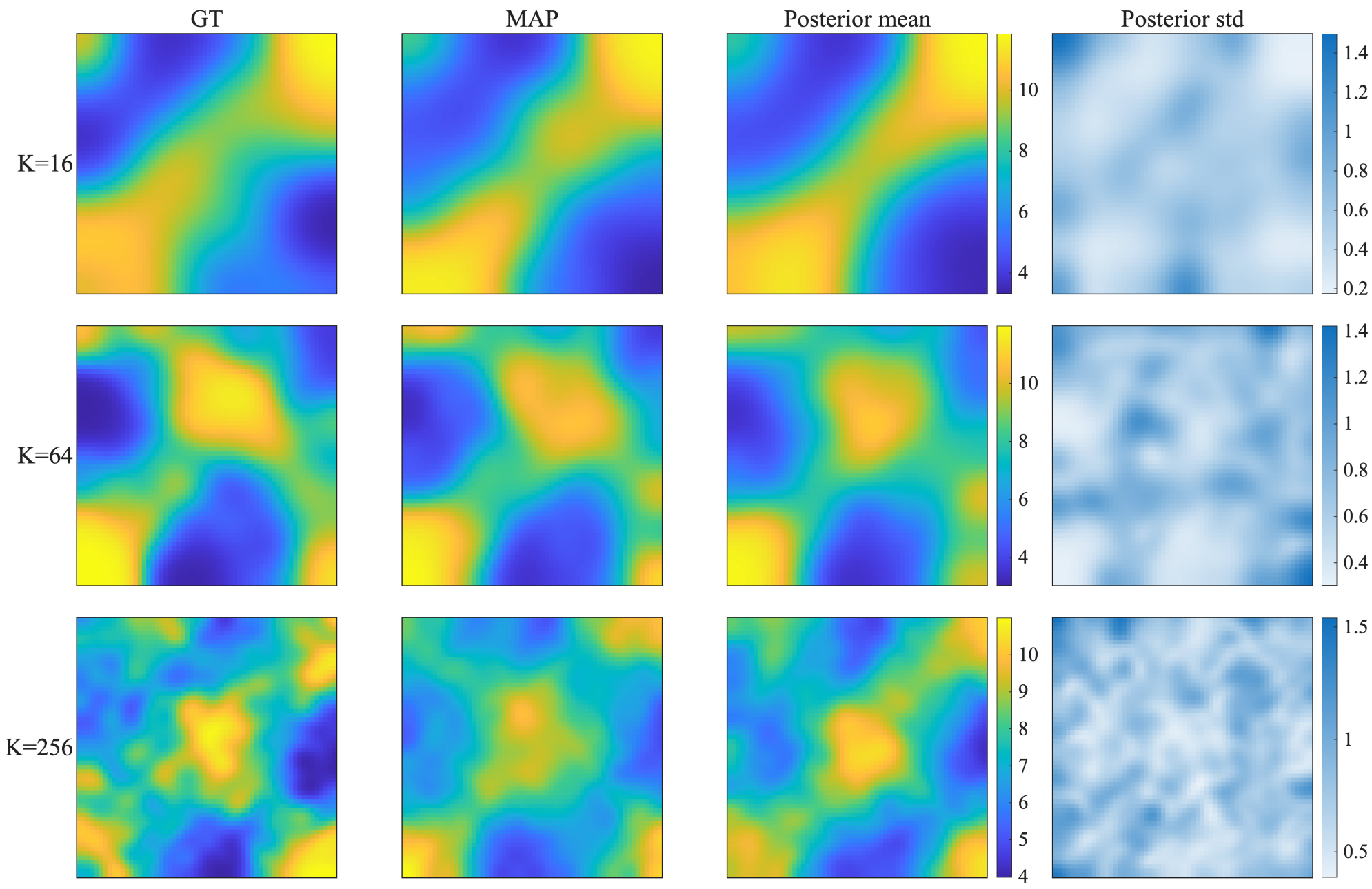

Supplementary Figure 7: Posterior inference of the spatially varying diffusion coefficient $D(\mathbf{x})$ for the 2D Darcy flow problem with KL truncation $K \in \{16, 64, 256\}$ (rows). Columns from left to right: ground truth $D(\mathbf{x})$, MAP estimate, posterior mean, and posterior standard deviation (note the different color scale on the right). Columns 1–3 share a common per-row color scale.

across the high-dimensional parameter. This is more conservative than the multivariate ESS. For example, for $K = 16$, the median ESS across coordinates is 6.8, while the multivariate ESS is 52.2.

K	sec / lower steps	average lower steps	median ESS	sec / ESS
16	0.066	10.2	6.8	954
64	0.068	36.7	7.0	1,205
256	0.059	564.4	6.2	4,804

Supplementary Table 3: Scalability of B-BiLO with the KL truncation dimension K for the 2D Darcy flow inverse problem. Wall-clock measured on a single GPU over $N = 999$ HMC iterations.

Two trends emerge from Table 3. First, the median ESS is comparable across K, indicating that the upper-level HMC sampling itself does not degrade markedly with dimension over this range. Second, the per-lower-step cost is essentially independent of

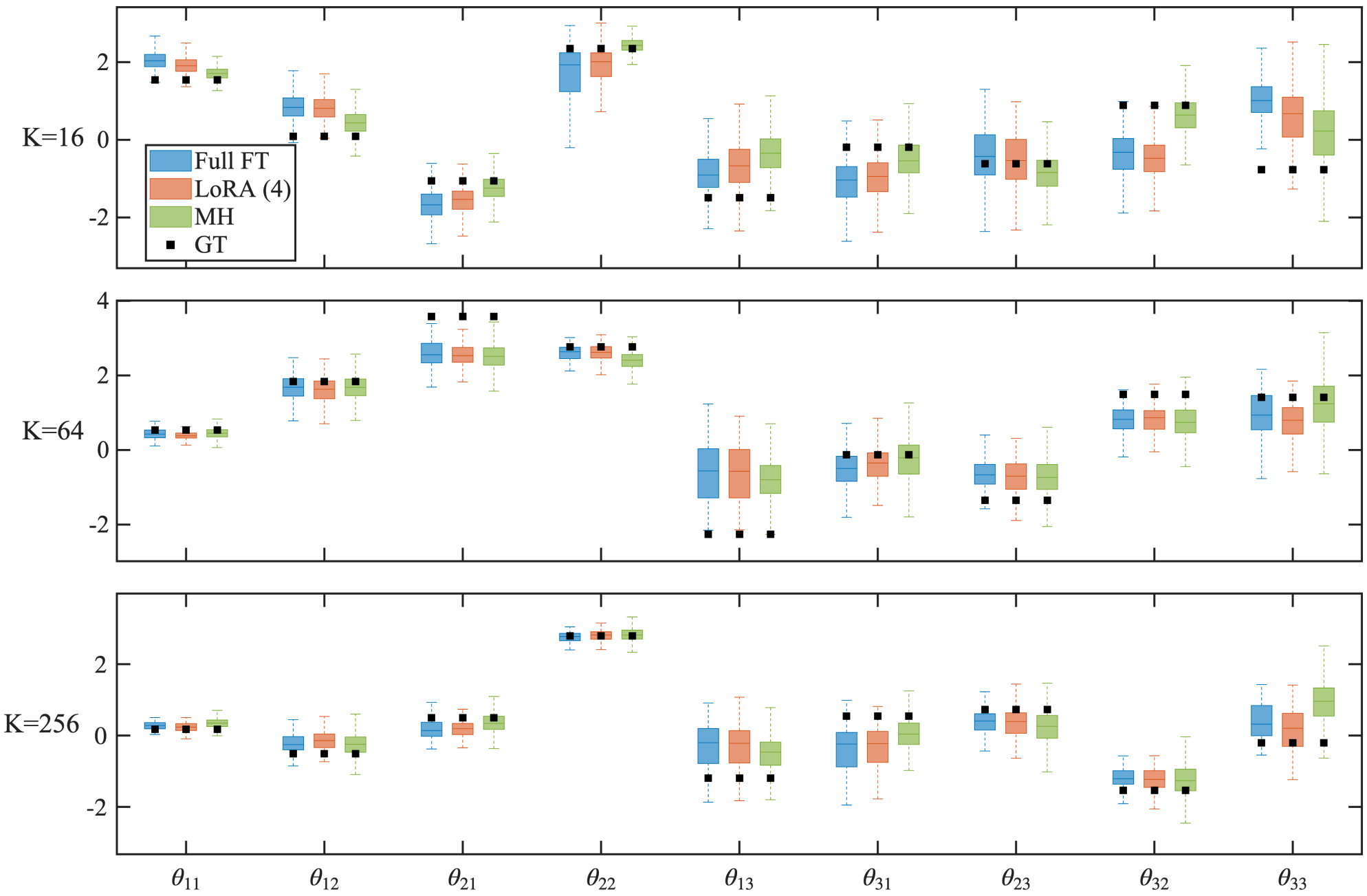

Supplementary Figure 8: Posterior box plots of the leading nine KL coefficients θ_{ij} (with small i, j) for the 2D Darcy flow problem with KL truncation $K \in \{16, 64, 256\}$ (rows). Each panel compares B-BiLO with full fine-tuning, B-BiLO with LoRA rank 4, and the reference MH sampler with a fine-grid numerical solver. Black squares mark the ground-truth coefficient values used to generate the data.

K. This is because the auxiliary-variable formulation differentiates with respect to the scalar $z = D(\mathbf{x}; \theta)$ rather than θ directly, the computational cost mainly depends on the number of residual points and the size of the network u. Third, the average number of lower-level fine-tuning steps per HMC iteration grows with K. This is the principal driver of the increase in the total wall-clock cost: as K grows, the displacement of θ along each leapfrog trajectory grows in magnitude, and the function $D(\mathbf{x}; \theta)$ becomes more oscillatory, so the lower-level optimization requires more steps to recover the the same lower level loss tolerance. Overall, the wall-clock per effective sample grows by roughly 5 times when K is increased by 16 times, demonstrating that B-BiLO remains practical for moderately high-dimensional function inference, while also identifying the lower-level convergence cost as the principal bottleneck for further scaling.

Supplementary References

[1] Zhang, R.Z., Miles, C.E., Xie, X., Lowengrub, J.S.: BiLO: Bilevel Local Operator Learning for PDE Inverse Problems. Journal of Computational Physics **551**, 114679 (2026) https://doi.org/10.1016/j.jcp.2026.114679

[2] Dashti, M., Stuart, A.M.: The Bayesian Approach To Inverse Problems. arXiv

(2015). https://doi.org/10.48550/arXiv.1302.6989

[3] Cleary, E., Garbuno-Inigo, A., Lan, S., Schneider, T., Stuart, A.M.: Calibrate, emulate, sample. Journal of Computational Physics **424**, 109716 (2021) https://doi.org/10.1016/j.jcp.2020.109716

[4] Neal, R.M.: MCMC Using Hamiltonian Dynamics. In: Handbook of Markov Chain Monte Carlo. Chapman and Hall/CRC

[5] Rudolf, D., Schweizer, N.: Perturbation theory for Markov chains via Wasserstein distance. Bernoulli **24**(4A), 2610–2639 (2018) https://doi.org/10.3150/17-BEJ938

[6] Chen, Z., Vempala, S.S.: Optimal Convergence Rate of Hamiltonian Monte Carlo for Strongly Logconcave Distributions. Theory of Computing **18**(9), 1–18 (2022) https://doi.org/10.4086/toc.2022.v018a009

[7] Yang, L., Meng, X., Karniadakis, G.E.: B-PINNs: Bayesian physics-informed neural networks for forward and inverse PDE problems with noisy data. Journal of Computational Physics **425**, 109913 (2021) https://doi.org/10.1016/j.jcp.2020.109913

[8] Rathore, P., Lei, W., Frangella, Z., Lu, L., Udell, M.: Challenges in Training PINNs: A Loss Landscape Perspective. arXiv (2024). https://doi.org/10.48550/arXiv.2402.01868

[9] Hastings, W.K.: Monte Carlo Sampling Methods Using Markov Chains and Their Applications. Biometrika **57**(1), 97–109 (1970) https://doi.org/10.2307/2334940 2334940

[10] Hoffman, M.D., Gelman, A.: The No-U-Turn Sampler: Adaptively Setting Path Lengths in Hamiltonian Monte Carlo. Journal of Machine Learning Research **15**(47), 1593–1623 (2014)

[11] Hernández-Sánchez, M., Kitaura, F.-S., Ata, M., Vecchia, C.D.: Higher Order Hamiltonian Monte Carlo Sampling for Cosmological Large-Scale Structure Analysis. Monthly Notices of the Royal Astronomical Society **502**(3), 3976–3992 (2021) https://doi.org/10.1093/mnras/stab123 arXiv:1911.02667 [astro-ph]

[12] Chen, T., Fox, E.B., Guestrin, C.: Stochastic Gradient Hamiltonian Monte Carlo. arXiv (2014). https://doi.org/10.48550/arXiv.1402.4102

[13] Zhang, R.Z., Ezhov, I., Balcerak, M., Zhu, A., Wiestler, B., Menze, B., Lowengrub, J.S.: Personalized predictions of Glioblastoma infiltration: Mathematical models, Physics-Informed Neural Networks and multimodal scans. Medical Image Analysis **101**, 103423 (2025) https://doi.org/10.1016/j.media.2024.103423

[14] Lipková, J., Angelikopoulos, P., Wu, S., Alberts, E., Wiestler, B., Diehl, C., Preibisch, C., Pyka, T., Combs, S.E., Hadjidoukas, P., Van Leemput, K., Koumoutsakos, P., Lowengrub, J., Menze, B.: Personalized Radiotherapy Design for Glioblastoma: Integrating Mathematical Tumor Models, Multimodal Scans, and Bayesian Inference. IEEE Transactions on Medical Imaging **38**(8), 1875–1884 (2019) https://doi.org/10.1109/TMI.2019.2902044

[15] Balcerak, M., Weidner, J., Karnakov, P., Ezhov, I., Litvinov, S., Koumoutsakos, P., Amiranashvili, T., Zhang, R.Z., Lowengrub, J.S., Yakushev, I., Wiestler, B., Menze, B.: Individualizing glioma radiotherapy planning by optimization of a data and physics-informed discrete loss. Nature Communications **16**(1), 1–16 (2025) https://doi.org/10.1038/s41467-025-60366-4

[16] Lim, K.L., Dutta, R., Rotaru, M.: Physics Informed Neural Network using Finite Difference Method. In: 2022 IEEE International Conference on Systems, Man, and Cybernetics (SMC), pp. 1828–1833 (2022). https://doi.org/10.1109/SMC53654.2022.9945171

[17] Chiu, P.-H., Wong, J.C., Ooi, C., Dao, M.H., Ong, Y.-S.: CAN-PINN: A fast physics-informed neural network based on coupled-automatic–numerical differentiation method. Computer Methods in Applied Mechanics and Engineering **395**, 114909 (2022) https://doi.org/10.1016/j.cma.2022.114909

[18] Kingma, D.P., Ba, J.: Adam: A method for stochastic optimization. In: 3rd International Conference on Learning Representations, ICLR 2015, San Diego, CA, USA, May 7-9, 2015, Conference Track Proceedings (2015). http://arxiv.org/abs/1412.6980

[19] Reddi, S.J., Kale, S., Kumar, S.: On the Convergence of Adam and Beyond. arXiv (2019). https://doi.org/10.48550/arXiv.1904.09237

[20] Wang, S., Teng, Y., Perdikaris, P.: Understanding and Mitigating Gradient Flow Pathologies in Physics-Informed Neural Networks. SIAM Journal on Scientific Computing **43**(5), 3055–3081 (2021) https://doi.org/10.1137/20M1318043

[21] Wang, S., Wang, H., Perdikaris, P.: On the eigenvector bias of Fourier feature networks: From regression to solving multi-scale PDEs with physics-informed neural networks. Computer Methods in Applied Mechanics and Engineering **384**, 113938 (2021) https://doi.org/10.1016/j.cma.2021.113938

[22] Tseng, Y.-H., Lin, T.-S., Hu, W.-F., Lai, M.-C.: A cusp-capturing PINN for elliptic interface problems. Journal of Computational Physics **491**, 112359 (2023) https://doi.org/10.1016/j.jcp.2023.112359

[23] He, K., Zhang, X., Ren, S., Sun, J.: Deep residual learning for image recognition. In: 2016 IEEE Conference on Computer Vision and Pattern Recognition, CVPR 2016, pp. 770–778 (2016). https://doi.org/10.1109/CVPR.2016.90 .

https://doi.org/10.1109/CVPR.2016.90

[24] Miles, C.E., McKinley, S.A., Ding, F., Lehoucq, R.B.: Inferring Stochastic Rates from Heterogeneous Snapshots of Particle Positions. Bulletin of Mathematical Biology **86**(6), 74 (2024) https://doi.org/10.1007/s11538-024-01301-4

[25] Huang, D.Z., Huang, J., Reich, S., Stuart, A.M.: Efficient derivative-free Bayesian inference for large-scale inverse problems. Inverse Problems **38**(12), 125006 (2022) https://doi.org/10.1088/1361-6420/ac99fa

[26] Li, Z., Huang, D.Z., Liu, B., Anandkumar, A.: Fourier neural operator with learned deformations for pdes on general geometries. J. Mach. Learn. Res. **24**, 388–138826 (2023)

[27] Vats, D., Flegal, J.M., Jones, G.L.: Multivariate output analysis for Markov chain Monte Carlo. Biometrika **106**(2), 321–337 (2019) https://doi.org/10.1093/biomet/asz002

[28] Geyer, C.J.: Practical Markov Chain Monte Carlo. Statistical Science **7**(4), 473–483 (1992) https://doi.org/10.1214/ss/1177011137
