# Supplementary material for: Bayesian bilevel operator learning with low-rank adaptation for efficient uncertainty quantification of PDE inverse problems
Source: Nat Commun. 2026 Sep 19;17:10979. doi: 10.1038/s41467-026-77768-7 (PMC13683411; doi:10.1038/s41467-026-77768-7)
Supplement: Supplementary file 2 [file 41467_2026_77768_MOESM2_ESM.pdf]

nature portfolio

Peer Review File

Bayesian Bilevel Operator Learning with Low-Rank Adaptation for Efficient Uncertainty Quantification of PDE Inverse Problems

Corresponding Author: Professor Ray Zirui Zhang

This file contains all reviewer reports in order by version, followed by all author rebuttals in order by version.

Version 0:

Reviewer comments:

Reviewer #1

(Remarks to the Author)
I do not believe this manuscript meets the bar of Nature Communications. While the paper proposes a technically sound framework and presents several numerical examples (some with real data), the central methodological contribution is not sufficiently validated, and the theoretical and empirical evidence do not fully support the claims. For a journal of this level, I would expect clearer guarantees of correctness, stronger comparative positioning relative to established methods, and more rigorous theoretical results.

First, the sampling methodology lacks a fully convincing justification of correctness. The paper integrates lower-level optimization within an HMC framework but does not rigorously establish that the resulting Markov chain preserves the intended posterior distribution. Given that the main claim is efficient Bayesian uncertainty quantification, this gap is significant and affects the soundness of the approach.

Second, the empirical evaluation is not sufficiently comprehensive to support the claims of efficiency and robustness. The comparisons focus primarily on Bayesian PINNs, without benchmarking against well-established methods in Bayesian PDE inversion (e.g., adjoint-based HMC or other mature approaches). Without these comparisons and more complete MCMC diagnostics, it is difficult to assess whether the method represents a substantive advance.

Third, the theoretical results appear relatively weak given the scope of the claims. The analysis mainly provides perturbation bounds under restrictive assumptions and does not deliver a rigorous guarantee of sampling correctness or convergence to a well-defined posterior. For a journal of this standard, one would expect stronger theoretical support, for example, either a clear invariance or convergence result for the algorithm or a more precise characterization of the bias and error introduced by the approximations.

For these reasons, I would not recommend publication in its present form.

(Remarks on code availability)

Reviewer #2

(Remarks to the Author)
I co-reviewed this manuscript with one of the reviewers who provided the listed reports. This is part of the Nature Communications initiative to facilitate training in peer review and to provide appropriate recognition for Early Career Researchers who co-review manuscripts.

(Remarks on code availability)

Reviewer #3

(Remarks to the Author)

This paper proposes Bayesian Bilevel Local Operator Learning (B-BiLO) for uncertainty quantification in PDE inverse problems. The method combines a bilevel formulation in which a neural network approximates a local PDE solution operator with Hamiltonian Monte Carlo (HMC) sampling over the physical parameters. The neural network weights are optimized deterministically, while only the physical parameters are sampled. Low-Rank Adaptation (LoRA) is introduced to reduce the cost of the lower-level optimization.

The framework is conceptually interesting. However, several practical aspects of the method require further clarification and validation. The results presented in the manuscript are promising, but I have the following comments and questions.

1. Robustness of the lower-level optimization
The method relies on repeatedly solving a lower-level optimization problem that enforces both the PDE residual and a residual-gradient condition. This step may be difficult for complex PDEs and could become a computational bottleneck. The manuscript does not report convergence behavior, iteration counts, or sensitivity to the residual-gradient weighting parameter. Additional empirical evidence demonstrating the robustness and computational cost of this optimization would be helpful.

2. Assumption B.1
The theoretical analysis assumes that the approximated PDE solution is Lipschitz continuous with respect to both the physical parameters and the neural network weights, and that parameter sensitivities are bounded. While similar smoothness assumptions are common in classical inverse problem theory for the parameter-to-solution map, the additional regularity assumption with respect to the neural network weights W is less standard and may be difficult to guarantee in practice. Since the neural solution depends on an optimization process, it would be helpful to discuss how these assumptions relate to the behavior of the trained neural solver in realistic settings.

3.In Fig. 2, the posterior mean of k by the proposed method appears to be around 0.75, while the ground truth parameter is k = 0.7, as stated on Page 8. It seems that the reference solution and other baseline methods also exhibit a similar bias. It would be helpful for the authors to explain the source of this discrepancy and clarify whether it arises from data noise, model mismatch, or solver approximation error.

4. In Section 4.5, the method is extended to infer unknown functions using a neural parameterization. As the number of trainable parameters increases, the dimension of the inferred parameter space becomes large, which may significantly increase the difficulty of Bayesian sampling. It is therefore unclear whether the proposed framework remains practical in such high-dimensional settings. Additional discussion or experiments addressing scalability would strengthen the paper.

(Remarks on code availability)

Reviewer #4

(Remarks to the Author)
Overall, the proposed methodology is interesting, novel, and applied to a reasonable array of problems relevant to the greater scientific community. However,
- the paper lacks rigor and clarity of mathematical and statistical presentation;
- overly relies on comparisons to BNNs which is not an accepted baseline for the chosen problems;
- poorly evaluates the benefits of the proposed method beyond showing that the method "works" on the selected problems.

For a journal with high standards, I would expect to see rigorously demonstrated benefits of each component of the proposed method, from the concurrent learning of a surrogate model to the addition of the derivative w.r.t. theta in the physics loss, and the low-rank update scheme. Proper community-accepted baselines should also be chosen for comparison, ex: a surrogate model pre-trained from parameter samples drawn from the prior (to show the benefit of concurrent surrogate learning), or a Calibrate Emulate Sample style scheme.

This work has good potential to have strong impact and should be pursued, but in the current state of the manuscript I do not believe it is ready for publication in a high-standards journal.

Further comments:

i) An important reference missing is:

Cleary, E., Garbuno-Inigo, A., Lan, S., Schneider, T. and Stuart, A.M., 2021. Calibrate, emulate, sample. Journal of Computational Physics, 424, p.109716.

I suggest the reviewers discuss/contrast their proposed method with this paper as there are many strong parallels. This is far more relevant of a comparison then BNNs. Furthermore, I suggest the authors borrow notation from this paper to improve the clarity of their work.

This paper is also highly relevant:

Yan, L. and Zhou, T., 2021. Stein variational gradient descent with local approximations. Computer Methods in Applied Mechanics and Engineering, 386, p.114087.

Overall the literature survey on Bayesian statistics with surrogate models is not developed enough.

ii) page 5. Calling "u(. , \theta)" a local operator is highly nonstandard as this is a function. Can this terminology be motivated properly, or revised. Is this meant to represent the map \theta\mapsto u taking parameters to PDE solutions on \Omega? If so please make this clear.

iii) Section 4.3 entitled theoretical analysis is far too terse and short to constitute a proper theoretical analysis of the method. I would ask if this can be re-labelled to directly reflect the content presented ("Some Theoretical Error Bounds" for example).

v) Contribution 2 on page 4 is not formulated as a contribution; it highlights the disadvantage of other methods. Also, BNNs only seem very loosely related to the aims of this paper, BNN produce uncertainty estimates over the solution field, whereas the proposed method centers on producing uncertainties on the parameters; there are distinct enough objective that I fail to see the purpose of laboring such comparisons when it is not a "gold-standard" method.

iv) Overall the paper lacks clarity and rigor of exposition, the notation is not idiomatic and in many parts unclear.

Minor comments:

page 3: "Additionally, variants of Ensemble Kalman Inversion (EnKI) can efficiently
estimate the first two moments in Bayesian settings". Does EnKI recover the two first moments? I believe it only recovers a point estimate.

Can you explain the use of [.] vs (.) it seems quite inconsistently used.

(Remarks on code availability)

Version 1:

Reviewer comments:

Reviewer #1

(Remarks to the Author)
I thank the authors for their careful and substantial revision of the manuscript. The paper has improved considerably since the previous version. Several of my earlier concerns have been addressed satisfactorily, and I no longer see these as obstacles to publication.

(Remarks on code availability)
The authors have provided code that appears to be a useful companion to the paper. The implementation is reasonably well-organized and well-documented, and the available instructions seem sufficient for a reader to install the required dependencies and run the main examples.

I inspected the code but did not independently verify every experiment. Based on my review, the code appears to support the reproducibility of the manuscript's main numerical results and should be a useful resource for the community.

Reviewer #2

(Remarks to the Author)
I co-reviewed this manuscript with one of the reviewers who provided the listed reports. This is part of the Nature Communications initiative to facilitate training in peer review and to provide appropriate recognition for Early Career Researchers who co-review manuscripts.

(Remarks on code availability)

Reviewer #3

(Remarks to the Author)

The revised draft addresses most of my concerns, except for my final comment regarding the potential limitations in high-dimensional settings. The issue is not merely computational cost. In high-dimensional problems, HMC can be difficult to tune and may suffer from low acceptance rates, wasted computation, or divergent transitions. In this sense, if the dimension of the inferred known parameter space becomes large, the proposed method may face substantial practical challenges. I encourage the authors to discuss this limitation and its potential impact on the applicability of their approach.

(Remarks on code availability)

Open Access This Peer Review File is licensed under a Creative Commons Attribution 4.0 International License, which permits use, sharing, adaptation, distribution and reproduction in any medium or format, as long as you give appropriate credit to the original author(s) and the source, provide a link to the Creative Commons license, and indicate if changes were made.
In cases where reviewers are anonymous, credit should be given to 'Anonymous Referee' and the source.
The images or other third party material in this Peer Review File are included in the article's Creative Commons license, unless indicated otherwise in a credit line to the material. If material is not included in the article's Creative Commons license and your intended use is not permitted by statutory regulation or exceeds the permitted use, you will need to obtain permission directly from the copyright holder.
To view a copy of this license, visit https://creativecommons.org/licenses/by/4.0/

Response to Reviewer 1

In this document, questions from the reviewer are shown in blue, our responses are in black, and revisions to the manuscript are marked in red.

Summary of Changes

We thank all the reviewers for their constructive feedback. Below is a summary of the main changes made in the revised manuscript and supplementary material (SM).

1. **Expanded theoretical analysis.** The theoretical analysis (Sec. 4.3 and SM Sec. B) has been significantly strengthened. Standard Hamiltonian Monte Carlo (HMC) with the leapfrog integrator is volume preserving and time reversible, and together with the Metropolis–Hastings correction these properties guarantee that the chain samples the target distribution exactly. We carefully analyze how the lower-level optimization in B-BiLO affects these key properties. We show that the B-BiLO leapfrog step is exactly volume-preserving (Proposition 1), the position reverses exactly while the momentum error is $O(\delta t \cdot \epsilon)$ (Proposition 2), and from these we bound the error of the transition kernel between the B-BiLO chain and the ideal chain. By leveraging the perturbation theory for Markov chains, we show that the Wasserstein-1 distance between the θ-marginal stationary distribution of the B-BiLO chain and the ideal posterior is $O(\epsilon\, L\, \delta t)$ (Theorem 4). This result subsumes the previous results that only considered the static error resulting from inaccurate solution of the PDE.

2. **Standardized notation.** We revised the notation throughout the manuscript to improve clarity and accessibility. In the main text, all quantities are expressed as functions of finite-dimensional vectors. In particular, the neural network solution is $u(\mathbf{x}, \theta; W)$, where the semicolon separates the physical parameter θ from the neural network weight W. This is to highlight the distinct role of θ and W in the bilevel optimization process. Functionals, partial evaluation, and operator notations are only explained and used in the SM. Parentheses $(\cdot)$ denote functions of vectors, while brackets $[\cdot]$ are reserved for functionals or operators (which are used only in the SM). We also point out the connection between the functions of the form $u(\mathbf{x}, \theta)$ and the parameter-to-solution map $\mathcal{G} : \theta \mapsto u(\cdot, \theta)$ in SM.

3. **Additional baselines and comparisons.** We added a comparison against a pretrained neural-operator surrogate (DeepONet) in both in-distribution and out-of-distribution settings (SM Sec. E.1), representing the neural operator / surrogate-based Bayesian inference paradigm. We added a paragraph to highlight the distinction from a local surrogate: the local operator is evaluated only at the current parameter iterate θ_k. The core idea is to obtain the descent direction of the potential energy with respect to the PDE parameters θ_k; the resulting condition turns out to be learning a function that approximates the solution operator at θ_k. The reference "Calibrate, Emulate, Sample" (Cleary et al., 2021) and "Stein variational gradient descent with local approximations" (Yan and Zhou, 2021) are now cited.

4. **MCMC diagnostics and cross-validation.** We report the Gelman–Rubin $\hat{R}$ diagnostic from 4 independent chains for the nonlinear Poisson and point process problems (SM Figs. E2 and E6), and provide a systematic cross-validation study of the lower-level tolerance ϵ and the residual-gradient weight w_{rgrad} (SM Fig. E4), demonstrating robustness.

5. **Scalability study with the KL truncation dimension.** We added an empirical scalability study for the 2D Darcy flow problem in which the KL truncation dimension is varied as $K \in \{16, 64, 256\}$ (SM Sec. E.4, Figs. E7 and E8, Table E3). The study reports the wall-clock per HMC iteration, the average number of lower-level fine-tuning steps, the median effective sample size across the KL coefficients, and the wall-clock per effective sample. The posterior mean is close to the ground-truth diffusion field, and the marginal posteriors of the leading KL coefficients are consistent with the ground truth at all three values of K. The wall-clock per effective sample grows sub-linearly with K.

Reviewer's overall comment I do not believe this manuscript meets the bar of Nature Communications. While the paper proposes a technically sound framework and presents several numerical examples (some with real data), the central methodological contribution is not sufficiently validated, and the theoretical and empirical evidence do not fully support the claims. For a journal of this level, I would expect clearer guarantees of correctness, stronger comparative positioning relative to established methods, and more rigorous theoretical results.

First, the sampling methodology lacks a fully convincing justification of correctness. The paper integrates lower-level optimization within an HMC framework but does not rigorously establish that the resulting Markov chain preserves the intended posterior distribution. Given that the main claim is efficient Bayesian uncertainty quantification, this gap is significant and affects the soundness of the approach.

Second, the empirical evaluation is not sufficiently comprehensive to support the claims of efficiency and robustness. The comparisons focus primarily on Bayesian PINNs, without benchmarking against well-established methods in Bayesian PDE inversion (e.g., adjoint-based HMC or other mature approaches). Without these comparisons and more complete MCMC diagnostics, it is difficult to assess whether the method represents a substantive advance.

Third, the theoretical results appear relatively weak given the scope of the claims. The analysis mainly provides perturbation bounds under restrictive assumptions and does not deliver a rigorous guarantee of sampling correctness or convergence to a well-defined posterior. For a journal of this standard, one would expect stronger theoretical support, for example, either a clear invariance or convergence result for the algorithm or a more precise characterization of the bias and error introduced by the approximations.

For these reasons, I would not recommend publication in its present form.

Response: We thank the reviewer for the detailed assessment and for identifying areas where the manuscript needed strengthening. We have substantially revised the paper to address each of the three main concerns.

1. Sampling correctness. We agree that the original manuscript did not rigorously establish the sampling guarantees of B-BiLO. In the revised manuscript (Sec. 4.3 and SM Sec. B.2), we now provide a detailed theoretical analysis based on the Markov chain perturbation framework of Rudolf and Schweizer [1].

The correctness of standard HMC rests on three properties: (i) the leapfrog integrator is volume-preserving; (ii) the leapfrog integrator is time-reversible (up to momentum flip); and (iii) a Metropolis–Hastings (MH) acceptance step corrects for the energy error introduced by the discrete integrator. Together, these three properties guarantee that the chain leaves the target posterior invariant. B-BiLO retains all three components (see Algorithm 1 in the manuscript, which explicitly includes the MH step), but the lower-level optimization is solved only approximately, which perturbs properties (i)–(iii). We analyze each perturbation carefully:

- **Volume preservation** (Proposition 1 in SM): The B-BiLO leapfrog step is *exactly* volume-preserving in the (θ, r) phase space. The key observation is that the neural network weights W live outside the sampled (θ, r) space and enter only through gradient evaluations. Each leapfrog sub-step remains a shear map in (θ, r), so the Jacobian is triangular with determinant 1, regardless of how W is computed.

- **Approximate time-reversibility** (Proposition 2 in SM): Time-reversibility holds only approximately. The position θ reverses exactly, but the momentum accumulates an error of order $O(\delta t \cdot \epsilon)$ per leapfrog step, where ϵ is the lower-level optimization tolerance and δt is the step size, arising because the lower-level solver is not time-reversible.

- **One-step kernel perturbation** (Proposition 3 in SM): Combining the above, we bound the Wasserstein-1 distance between the ideal HMC transition kernel and the B-BiLO kernel by $O(\epsilon \cdot L \cdot \delta t)$, where L is the number of leapfrog steps.

Applying the perturbation theory of Rudolf and Schweizer to this one-step bound, we establish the following guarantee on the Wasserstein-1 distance between the θ-marginal stationary distribution $\bar{\pi}$ of the B-BiLO chain and the true posterior π^* (Theorem 4 in SM) $\mathcal{W}_1(\bar{\pi}, \pi^*) = O(\epsilon \cdot L \cdot \delta t)$.

2. Empirical evaluation and comparisons. We acknowledge that the original comparisons focused primarily on Bayesian PINNs. In the revised manuscript we have added the following comparisons and diagnostics:

- **Neural operator (surrogate) comparison**: We compare against a pretrained DeepONet surrogate for the parameter-to-solution map, in both in-distribution (ID) and out-of-distribution (OOD) settings (SM Sec. E.1, Fig. E3, Table E1). The surrogate yields a reasonable posterior in the ID case, but the OOD case produces a broader and less accurate posterior. B-BiLO avoids this degradation because it does not require a pretraining dataset and refines the operator locally at each θ during sampling, though at the cost of additional fine-tuning steps per sample.

- **Gelman–Rubin $\hat{R}$ diagnostic**: We report the $\hat{R}$ diagnostic computed from 4 independent chains for the nonlinear Poisson problem (SM Fig. E2) and the point process problem (SM Fig. E6). The $\hat{R}$ values are close to 1 in both cases, indicating good convergence and mixing.

- **Cross-validation of hyperparameters**: We provide a systematic study of the sensitivity to the lower-level tolerance ϵ and the residual-gradient weight w_{rgrad} (SM Fig. E4), showing that B-BiLO is robust to the choice of w_{rgrad} and that ϵ is the primary driver of both optimization accuracy and posterior quality.

Regarding the comparison with adjoint-based HMC: B-BiLO and adjoint-based HMC are conceptually related—both compute the gradient of the potential energy with respect to the parameters, $\nabla_\theta U(\theta)$, subject to the PDE constraint and use it for gradient-based MCMC. The key difference is that adjoint-based HMC requires deriving and implementing the adjoint PDE, which becomes challenging for complex PDE models (such as Example 2, the GBM tumor growth problem) and less flexible when experimenting with different PDE models. B-BiLO provides gradient-based MCMC without adjoint derivation or pretraining datasets. Within the design space of gradient-based methods with neural PDE solvers, BPINN is one of the most popular methods, and the comparison in Example 1 demonstrates that B-BiLO resolves the key accuracy–efficiency tension that limits BPINN. Across all examples, the reference solution obtained via Metropolis–Hastings with a high-accuracy numerical PDE solver serves as the accuracy benchmark, confirming that B-BiLO recovers the correct posterior.

3. Theoretical results. As described under point 1, the theoretical analysis has been significantly expanded. The original manuscript provided only a KL divergence bound ($D_{\mathrm{KL}}(\bar{\pi}\|\pi^*) = O(\epsilon)$) on the static target error arising from inexact PDE solution. The revised manuscript replaces this with a Wasserstein-1 bound on the θ-marginal stationary distribution of the B-BiLO Markov chain (Theorem 4 in SM), which directly characterizes the *sampling* error, accounts for the dynamics of the Markov chain, and establishes a clear, controllable link between the lower-level tolerance ϵ and the accuracy of the posterior samples.

References

[1] Rudolf, D., Schweizer, N.: Perturbation theory for Markov chains via Wasserstein distance. Bernoulli **24**(4A), 2610–2639 (2018) `https://doi.org/10.3150/17-BEJ938`

Response to Reviewer 2

In this document, questions from the reviewer are shown in blue, our responses are in black, and revisions to the manuscript are marked in red.

Summary of Changes

We thank all the reviewers for their constructive feedback. Below is a summary of the main changes made in the revised manuscript and supplementary material (SM).

1. **Expanded theoretical analysis.** The theoretical analysis (Sec. 4.3 and SM Sec. B) has been significantly strengthened. Standard Hamiltonian Monte Carlo (HMC) with the leapfrog integrator is volume preserving and time reversible, and together with the Metropolis–Hastings correction these properties guarantee that the chain samples the target distribution exactly. We carefully analyze how the lower-level optimization in B-BiLO affects these key properties. We show that the B-BiLO leapfrog step is exactly volume-preserving (Proposition 1), the position reverses exactly while the momentum error is $O(\delta t \cdot \epsilon)$ (Proposition 2), and from these we bound the error of the transition kernel between the B-BiLO chain and the ideal chain. By leveraging the perturbation theory for Markov chains, we show that the Wasserstein-1 distance between the θ-marginal stationary distribution of the B-BiLO chain and the ideal posterior is $O(\epsilon\, L\, \delta t)$ (Theorem 4). This result subsumes the previous results that only considered the static error resulting from inaccurate solution of the PDE.

2. **Standardized notation.** We revised the notation throughout the manuscript to improve clarity and accessibility. In the main text, all quantities are expressed as functions of finite-dimensional vectors. In particular, the neural network solution is $u(\mathbf{x}, \theta; W)$, where the semicolon separates the physical parameter θ from the neural network weight W. This is to highlight the distinct role of θ and W in the bilevel optimization process. Functionals, partial evaluation, and operator notations are only explained and used in the SM. Parentheses $(\cdot)$ denote functions of vectors, while brackets $[\cdot]$ are reserved for functionals or operators (which are used only in the SM). We also point out the connection between the functions of the form $u(\mathbf{x}, \theta)$ and the parameter-to-solution map $\mathcal{G} : \theta \mapsto u(\cdot, \theta)$ in SM.

3. **Additional baselines and comparisons.** We added a comparison against a pretrained neural-operator surrogate (DeepONet) in both in-distribution and out-of-distribution settings (SM Sec. E.1), representing the neural operator / surrogate-based Bayesian inference paradigm. We added a paragraph to highlight the distinction from a local surrogate: the local operator is evaluated only at the current parameter iterate θ_k. The core idea is to obtain the descent direction of the potential energy with respect to the PDE parameters θ_k; the resulting condition turns out to be learning a function that approximates the solution operator at θ_k. The reference "Calibrate, Emulate, Sample" (Cleary et al., 2021) and "Stein variational gradient descent with local approximations" (Yan and Zhou, 2021) are now cited.

4. **MCMC diagnostics and cross-validation.** We report the Gelman–Rubin $\hat{R}$ diagnostic from 4 independent chains for the nonlinear Poisson and point process problems (SM Figs. E2 and E6), and provide a systematic cross-validation study of the lower-level tolerance ϵ and the residual-gradient weight w_{rgrad} (SM Fig. E4), demonstrating robustness.

5. **Scalability study with the KL truncation dimension.** We added an empirical scalability study for the 2D Darcy flow problem in which the KL truncation dimension is varied as $K \in \{16, 64, 256\}$ (SM Sec. E.4, Figs. E7 and E8, Table E3). The study reports the wall-clock per HMC iteration, the average number of lower-level fine-tuning steps, the median effective sample size across the KL coefficients, and the wall-clock per effective sample. The posterior mean is close to the ground-truth diffusion field, and the marginal posteriors of the leading KL coefficients are consistent with the ground truth at all three values of K. The wall-clock per effective sample grows sub-linearly with K.

Reviewer's overall comment I co-reviewed this manuscript with one of the reviewers who provided the listed reports. This is part of the Nature Communications initiative to facilitate training in peer review and to provide appropriate recognition for Early Career Researchers who co-review manuscripts.

Response: Thank you for your contribution to the review process and for taking the time to review our manuscript.

Response to Reviewer 3

In this document, questions from the reviewer are shown in blue, our responses are in black, and revisions to the manuscript are marked in red.

Summary of Changes

We thank all the reviewers for their constructive feedback. Below is a summary of the main changes made in the revised manuscript and supplementary material (SM).

1. **Expanded theoretical analysis.** The theoretical analysis (Sec. 4.3 and SM Sec. B) has been significantly strengthened. Standard Hamiltonian Monte Carlo (HMC) with the leapfrog integrator is volume preserving and time reversible, and together with the Metropolis–Hastings correction these properties guarantee that the chain samples the target distribution exactly. We carefully analyze how the lower-level optimization in B-BiLO affects these key properties. We show that the B-BiLO leapfrog step is exactly volume-preserving (Proposition 1), the position reverses exactly while the momentum error is $O(\delta t \cdot \epsilon)$ (Proposition 2), and from these we bound the error of the transition kernel between the B-BiLO chain and the ideal chain. By leveraging the perturbation theory for Markov chains, we show that the Wasserstein-1 distance between the θ-marginal stationary distribution of the B-BiLO chain and the ideal posterior is $O(\epsilon\, L\, \delta t)$ (Theorem 4). This result subsumes the previous results that only considered the static error resulting from inaccurate solution of the PDE.

2. **Standardized notation.** We revised the notation throughout the manuscript to improve clarity and accessibility. In the main text, all quantities are expressed as functions of finite-dimensional vectors. In particular, the neural network solution is $u(\mathbf{x}, \theta; W)$, where the semicolon separates the physical parameter θ from the neural network weight W. This is to highlight the distinct role of θ and W in the bilevel optimization process. Functionals, partial evaluation, and operator notations are only explained and used in the SM. Parentheses $(\cdot)$ denote functions of vectors, while brackets $[\cdot]$ are reserved for functionals or operators (which are used only in the SM). We also point out the connection between the functions of the form $u(\mathbf{x}, \theta)$ and the parameter-to-solution map $\mathcal{G} : \theta \mapsto u(\cdot, \theta)$ in SM.

3. **Additional baselines and comparisons.** We added a comparison against a pretrained neural-operator surrogate (DeepONet) in both in-distribution and out-of-distribution settings (SM Sec. E.1), representing the neural operator / surrogate-based Bayesian inference paradigm. We added a paragraph to highlight the distinction from a local surrogate: the local operator is evaluated only at the current parameter iterate θ_k. The core idea is to obtain the descent direction of the potential energy with respect to the PDE parameters θ_k; the resulting condition turns out to be learning a function that approximates the solution operator at θ_k. The reference "Calibrate, Emulate, Sample" (Cleary et al., 2021) and "Stein variational gradient descent with local approximations" (Yan and Zhou, 2021) are now cited.

4. **MCMC diagnostics and cross-validation.** We report the Gelman–Rubin $\hat{R}$ diagnostic from 4 independent chains for the nonlinear Poisson and point process problems (SM Figs. E2 and E6), and provide a systematic cross-validation study of the lower-level tolerance ϵ and the residual-gradient weight w_{rgrad} (SM Fig. E4), demonstrating robustness.

5. **Scalability study with the KL truncation dimension.** We added an empirical scalability study for the 2D Darcy flow problem in which the KL truncation dimension is varied as $K \in \{16, 64, 256\}$ (SM Sec. E.4, Figs. E7 and E8, Table E3). The study reports the wall-clock per HMC iteration, the average number of lower-level fine-tuning steps, the median effective sample size across the KL coefficients, and the wall-clock per effective sample. The posterior mean is close to the ground-truth diffusion field, and the marginal posteriors of the leading KL coefficients are consistent with the ground truth at all three values of K. The wall-clock per effective sample grows sub-linearly with K.

Reviewer's overall comment This paper proposes Bayesian Bilevel Local Operator Learning (B-BiLO) for uncertainty quantification in PDE inverse problems. The method combines a bilevel formulation in which a neural network approximates a local PDE solution operator with Hamiltonian Monte Carlo (HMC) sampling over the physical parameters. The neural network weights are optimized deterministically, while only the physical parameters are sampled. Low-Rank Adaptation (LoRA) is introduced to reduce the cost of the lower-level optimization.

The framework is conceptually interesting. However, several practical aspects of the method require further clarification and validation. The results presented in the manuscript are promising, but I have the following comments and questions.

Response: We thank the reviewer for the positive assessment of the framework and for the constructive comments. We have addressed each point below and made corresponding revisions to the manuscript and supplementary material.

Reviewer's specific question 1: 1. Robustness of the lower-level optimization The method relies on repeatedly solving a lower-level optimization problem that enforces both the PDE residual and a residual-gradient condition. This step may be difficult for complex PDEs and could become a computational bottleneck. The manuscript does not report convergence behavior, iteration counts, or sensitivity to the residual-gradient weighting parameter. Additional empirical evidence demonstrating the robustness and computational cost of this optimization would be helpful.

Response: We agree that this is an important practical concern. In the revised supplementary material (SM Sec. E.1, Fig. E4), we now include a systematic cross-validation study for the nonlinear Poisson problem that reports:

- The residual loss L_{res} and residual-gradient loss L_{rgrad} as a function of the stopping tolerance ϵ;

- The average number of lower-level iterations per HMC step;
- The resulting posterior mean and standard deviation of the parameter k, and the posterior standard deviation of $u(0)$ (as a proxy for solution uncertainty);

each for several values of the residual-gradient weight $w_{\mathrm{rgrad}} \in 10^{-3}, 10^{-2}, 10^{-1}$.

The key findings are: (1) the lower-level tolerance ϵ is the primary driver of both optimization accuracy and posterior quality—decreasing ϵ from 10^{-3} to 10^{-5} consistently reduces both losses, drives the posterior closer to the reference, and decreases posterior spread, at the cost of more iterations; (2) the method is relatively robust to the choice of w_{rgrad}: for all three values tested, the posterior mean and standard deviation are comparable, though larger w_{rgrad} provides stronger suppression of L_{rgrad} at the cost of more iterations; (3) the two losses are not in tension, since for a local operator both vanish simultaneously. We note that the lower-level problem is a standard neural-network training problem (minimizing residuals on collocation points), which benefits from warm-starting: each new θ in the HMC trajectory is close to the previous one, so convergence is typically fast (a few to tens of iterations rather than training from scratch). This is the central benefit of the bilevel structure with fine-tuning.

Reviewer's specific question 2: 2. Assumption B.1 The theoretical analysis assumes that the approximated PDE solution is Lipschitz continuous with respect to both the physical parameters and the neural network weights, and that parameter sensitivities are bounded. While similar smoothness assumptions are common in classical inverse problem theory for the parameter-to-solution map, the additional regularity assumption with respect to the neural network weights W is less standard and may be difficult to guarantee in practice. Since the neural solution depends on an optimization process, it would be helpful to discuss how these assumptions relate to the behavior of the trained neural solver in realistic settings.

Response: We thank the reviewer for prompting us to revisit this assumption. We have corrected Assumption B.1 in the revised SM (Sec. B.1): Lipschitz continuity of u with respect to W is not required. The analysis operates in the abstract function space and bounds the error of the approximate gradient in terms of the closeness of $\bar{u}$ (approximate solution of the PDE) to the true PDE solution u—a property governed entirely by the PDE stability and smoothness assumptions (Assumption B.1(ii)–(iii)). The parameterization of u (neural network, finite element basis, etc.) enters only through the inexact minimization assumption (B.1(i)): as long as one can find weights $\bar{W}$ such that $\mathcal{L}_{\mathrm{LO}}(\theta, \bar{W}) \leq \epsilon$, the gradient error bound holds regardless of how u is parameterized. In practice, achieving (i) benefits from active research on designing effective neural network architectures, optimization algorithms, and training strategies for solving PDEs with neural networks.

Reviewer's specific question 3: 3. In Fig. 2, the posterior mean of k by the proposed method appears to be around 0.75, while the ground truth parameter is k = 0.7, as stated on Page 8. It seems that the reference solution and other baseline methods also exhibit a similar bias. It would be helpful for the authors to explain the source of this discrepancy and clarify whether it arises from data noise, model mismatch, or solver approximation error.

Response: Thank you for this observation. The discrepancy between the posterior mean ($\approx$0.75) and the ground truth ($k = 0.7$) is due to the particular noise realization, not model mismatch or solver approximation error. In particular:

- The reference solution, obtained by Metropolis–Hastings sampling with a highly accurate numerical solver (finite difference on a fine grid), also yields a posterior mean of approximately 0.754 (see Table E1 in the SM). This confirms that the shift is not caused by the B-BiLO solver or neural network approximation.
- The noisy data are generated by adding i.i.d. Gaussian noise to the true solution at only 6 observation points. With such sparse and noisy observations, a single noise draw can noticeably shift the posterior. The posterior standard deviation of k is about 0.025, so the ground truth $k = 0.7$ lies within approximately 2 standard deviations of the posterior mean, which is consistent with the noise level.
- All methods (B-BiLO, BPINN, and reference) exhibit a similar shift, further confirming that this is a data-driven effect rather than a methodological artifact.

We have added a discussion of this point in the revised main text (Sec. 3.1):

"The posterior mean of k is approximately 0.75 across all methods, which differs from the ground truth $k^{\mathrm{GT}} = 0.7$. This shift is attributable to the particular noise realization: only 6 noisy observations are available, and with such sparse data, a single noise draw can noticeably shift the posterior. Indeed, the ground truth lies within approximately 2 standard deviations of the posterior mean ($\mathrm{std}(k) \approx 0.025$), and the reference solution obtained with a highly accurate numerical solver exhibits the same shift."

In addition, we have included a comparison against a pretrained neural-operator surrogate (DeepONet) in both in-distribution (ID) and out-of-distribution (OOD) settings (SM Sec. E.1, Fig. E3, Table E1). The surrogate gives accurate posterior estimates in the ID case, while the OOD case—where the ground truth lies outside the pretraining range—produces a broader and less accurate posterior, illustrating the sensitivity of surrogate-based approaches to distribution mismatch that B-BiLO avoids.

Reviewer's specific question 4: 4. In Section 4.5, the method is extended to infer unknown functions using a neural parameterization. As the number of trainable parameters increases, the dimension of the inferred parameter space becomes large, which may significantly increase the difficulty of Bayesian sampling. It is therefore unclear whether the proposed framework remains practical in such high-dimensional settings. Additional discussion or experiments addressing scalability would strengthen the paper.

Response: This is an important concern, and we have added an empirical scalability study to address it.

Built-in dimension reduction. B-BiLO already reduces the effective sampling dimension in two ways: (1) compared with Bayesian neural networks, the neural network weights are optimized deterministically rather than sampled, so only the physical parameters (e.g., the KL coefficients) enter the MCMC chain; (2) the auxiliary variable $z = f(\mathbf{x})$ reduces the

residual-gradient computation from differentiating through the full parameter vector $\theta \in \mathbb{R}^K$ to differentiating with respect to the scalar z, so the per-lower-step cost does not scale with $\dim \theta$.

New scalability experiment (KL truncation $K \in \{16, 64, 256\}$). We have rerun the Darcy flow inverse problem (Example 4) with the KL truncation increased from $K = 16$ to $K = 256$ (SM Sec. E.4, Figs. E7 and E8, Table E3). For each K, the ground truth is drawn from the corresponding K-dimensional KL prior and the inference is performed under the same prior, so the experiment isolates the effect of the inference dimension on sampling cost without any model-misspecification confound. The 2D posterior mean and standard deviation fields show that B-BiLO recovers a posterior mean and MAP that is close to the ground truth at all three values of K (Fig. E7); the marginal posteriors of the leading KL coefficients (Fig. E8) confirm that the ground-truth coefficients are well captured at all three resolutions, and that B-BiLO with LoRA rank 4 matches both full fine-tuning and a reference MH sampler with a fine-grid numerical solver. Quantitatively (Table E3), the per-lower-step cost is essentially independent of K (≈ 0.06–$0.07\,\mathrm{s}$) and the median effective sample size is comparable across K, while the average number of lower-level fine-tuning steps per HMC iteration grows from 10 to 564.

Two compounding effects explain this growth. First, with the leapfrog step size and number of leapfrog steps held fixed, the magnitude of the displacement of θ along each HMC trajectory grows with K (since the kinetic momentum is drawn from $\mathcal{N}(0, I_K)$, the per-trajectory ℓ^2 displacement scales as $\sqrt{K}$), so the function $D(\mathbf{x}; \theta)$ that the network must re-fit changes more between consecutive HMC iterates. Second, larger K admits higher-frequency KL eigenmodes, so $D(\mathbf{x}; \theta)$ becomes more oscillatory and is intrinsically harder for the network to fit to the same residual tolerance. Together, at the fixed tolerance ϵ, the lower-level optimization must close a larger gap to a more complex target, which requires more iterations.

The resulting wall-clock per effective sample grows by roughly $5\times$ when K is increased by $16\times$, indicating sub-linear cost growth and demonstrating that B-BiLO remains practical for moderately high-dimensional function inference. The study also identifies the lower-level convergence cost as the principal bottleneck for further scaling.

Path to higher dimensions. For scaling to substantially higher-dimensional settings, our framework is compatible in principle with mini-batch evaluation of the residual loss at collocation points and with stochastic-gradient samplers such as SGLD or stochastic-gradient HMC at the upper level, which we view as future extensions of the present work.

Response to Reviewer 4

In this document, questions from the reviewer are shown in blue, our responses are in black, and revisions to the manuscript are marked in red.

Summary of Changes

We thank all the reviewers for their constructive feedback. Below is a summary of the main changes made in the revised manuscript and supplementary material (SM).

1. **Expanded theoretical analysis.** The theoretical analysis (Sec. 4.3 and SM Sec. B) has been significantly strengthened. Standard Hamiltonian Monte Carlo (HMC) with the leapfrog integrator is volume preserving and time reversible, and together with the Metropolis–Hastings correction these properties guarantee that the chain samples the target distribution exactly. We carefully analyze how the lower-level optimization in B-BiLO affects these key properties. We show that the B-BiLO leapfrog step is exactly volume-preserving (Proposition 1), the position reverses exactly while the momentum error is $O(\delta t \cdot \epsilon)$ (Proposition 2), and from these we bound the error of the transition kernel between the B-BiLO chain and the ideal chain. By leveraging the perturbation theory for Markov chains, we show that the Wasserstein-1 distance between the θ-marginal stationary distribution of the B-BiLO chain and the ideal posterior is $O(\epsilon\, L\, \delta t)$ (Theorem 4). This result subsumes the previous results that only considered the static error resulting from inaccurate solution of the PDE.

2. **Standardized notation.** We revised the notation throughout the manuscript to improve clarity and accessibility. In the main text, all quantities are expressed as functions of finite-dimensional vectors. In particular, the neural network solution is $u(\mathbf{x}, \theta; W)$, where the semicolon separates the physical parameter θ from the neural network weight W. This is to highlight the distinct role of θ and W in the bilevel optimization process. Functionals, partial evaluation, and operator notations are only explained and used in the SM. Parentheses $(\cdot)$ denote functions of vectors, while brackets $[\cdot]$ are reserved for functionals or operators (which are used only in the SM). We also point out the connection between the functions of the form $u(\mathbf{x}, \theta)$ and the parameter-to-solution map $\mathcal{G} : \theta \mapsto u(\cdot, \theta)$ in SM.

3. **Additional baselines and comparisons.** We added a comparison against a pretrained neural-operator surrogate (DeepONet) in both in-distribution and out-of-distribution settings (SM Sec. E.1), representing the neural operator / surrogate-based Bayesian inference paradigm. We added a paragraph to highlight the distinction from a local surrogate: the local operator is evaluated only at the current parameter iterate θ_k. The core idea is to obtain the descent direction of the potential energy with respect to the PDE parameters θ_k; the resulting condition turns out to be learning a function that approximates the solution operator at θ_k. The reference "Calibrate, Emulate, Sample" (Cleary et al., 2021) and "Stein variational gradient descent with local approximations" (Yan and Zhou, 2021) are now cited.

4. **MCMC diagnostics and cross-validation.** We report the Gelman–Rubin $\hat{R}$ diagnostic from 4 independent chains for the nonlinear Poisson and point process problems (SM Figs. E2 and E6), and provide a systematic cross-validation study of the lower-level tolerance ϵ and the residual-gradient weight w_{rgrad} (SM Fig. E4), demonstrating robustness.

5. **Scalability study with the KL truncation dimension.** We added an empirical scalability study for the 2D Darcy flow problem in which the KL truncation dimension is varied as $K \in \{16, 64, 256\}$ (SM Sec. E.4, Figs. E7 and E8, Table E3). The study reports the wall-clock per HMC iteration, the average number of lower-level fine-tuning steps, the median effective sample size across the KL coefficients, and the wall-clock per effective sample. The posterior mean is close to the ground-truth diffusion field, and the marginal posteriors of the leading KL coefficients are consistent with the ground truth at all three values of K. The wall-clock per effective sample grows sub-linearly with K.

Reviewer's overall comment Overall, the proposed methodology is interesting, novel, and applied to a reasonable array of problems relevant to the greater scientific community. However, - the paper lacks rigor and clarity of mathematical and statistical presentation; - overly relies on comparisons to BNNs which is not an accepted baseline for the chosen problems; - poorly evaluates the benefits of the proposed method beyond showing that the method "works" on the selected problems.

For a journal with high standards, I would expect to see rigorously demonstrated benefits of each component of the proposed method, from the concurrent learning of a surrogate model to the addition of the derivative w.r.t. theta in the physics loss, and the low-rank update scheme. Proper community-accepted baselines should also be chosen for comparison, ex: a surrogate model pre-trained from parameter samples drawn from the prior (to show the benefit of concurrent surrogate learning), or a Calibrate Emulate Sample style scheme.

This work has good potential to have strong impact and should be pursued, but in the current state of the manuscript I do not believe it is ready for publication in a high-standards journal.

Response: We thank the reviewer for recognizing the novelty and potential impact of the work, and for the constructive criticism. We have made substantial revisions to address each of the concerns raised, including improved notation and exposition, expanded theoretical analysis, additional baselines, and new cross-validation experiments. We respond to each specific question below.

Reviewer's specific question 1: i) An important reference missing is:

Cleary, E., Garbuno-Inigo, A., Lan, S., Schneider, T. and Stuart, A.M., 2021. Calibrate, emulate, sample. Journal of Computational Physics, 424, p.109716.

I suggest the reviewers discuss/contrast their proposed method with this paper as there are many strong parallels. This is far more relevant of a comparison then BNNs. Furthermore, I suggest the authors borrow notation from this paper to improve the clarity of their work.

This paper is also highly relevant:

Yan, L. and Zhou, T., 2021. Stein variational gradient descent with local approximations. Computer Methods in Applied Mechanics and Engineering, 386, p.114087.

Overall the literature survey on Bayesian statistics with surrogate models is not developed enough.

Response: We thank the reviewer for pointing out these important references. We clarify how B-BiLO relates to and differs from CES and SVGD with local approximations, and we have expanded the surrogate literature survey in the introduction.

B-BiLO vs. surrogate-based methods. The Calibrate–Emulate–Sample (CES) framework [1] calibrates parameters via Ensemble Kalman Inversion, trains a Gaussian process emulator on the calibrated samples, and then replaces the forward model with the emulator for MCMC sampling. Yan & Zhou [2] build a local DNN surrogate of the parameter-to-observable map and evaluate it at all N SVGD particles at each step, with true-model evaluations added adaptively near the posterior to refine the surrogate over time. In both cases, the surrogates are queried at many parameter values during sampling, and their accuracy across parameter space directly determines posterior fidelity.

B-BiLO operates on a different principle. The local operator is trained concurrently with HMC; no offline calibration or data-generation phase is needed. The network is never evaluated at any parameter value other than the current HMC iterate θ_k. Its sole role is to provide a differentiable computational graph at θ_k so that automatic differentiation can compute $\nabla_\theta U(\theta_k)$ consistently with the PDE constraint. The term "local" in our method refers to this single-point consistency requirement (Conditions 1 and 2 enforced only at θ_k), not to a neighborhood interpolant. Because the network is never queried outside θ_k, there is no generalization requirement across parameter space, and out-of-distribution degradation does not arise. The local operator is therefore different from a "surrogate": it is not an approximation of the parameter-to-solution map evaluated at multiple points. We update the manuscript text to clarify this distinction (after defining the bilevel sampling problem):

Unlike surrogate (emulator) approaches, the local operator is never evaluated outside the current iterate θ: its role is to provide the gradient of the potential energy with respect to θ.

To further demonstrate the contrast with offline surrogates, we have added a comparison with a pretrained DeepONet surrogate in both in-distribution (ID) and out-of-distribution (OOD) settings (SM Sec. E.1, Fig. E3, Table E1), showing that the OOD case degrades the posterior, while B-BiLO avoids this issue, at the cost of concurrent training overhead.

Regarding the suggestion to borrow notation from the CES paper, we have partially adopted CES conventions where they improve clarity; we address this in detail in our response to question (iv) below.

Bayesian inference with surrogates. We have also expanded the surrogate literature paragraph in the introduction, which now reads as follows:

Surrogates (or emulators) approximate the parameter-to-solution map offline and, once trained, enable rapid evaluations for posterior sampling with either gradient-based or derivative-free methods [3, 4]. Gaussian process (GP) surrogates are the classical choice [5]: they work well for low-dimensional quantities of interest, require relatively few training samples, and provide built-in uncertainty quantification of the emulator itself. The Calibrate–Emulate–

Sample (CES) framework [1] is a canonical example—it calibrates parameters via Ensemble Kalman Inversion, trains a GP emulator on the calibrated samples, and then uses the emulator for MCMC sampling. For higher-dimensional parameter-to-solution maps involving full solution fields, neural surrogates scale better: these include CNN-based emulators [6] and physics-informed Bayesian neural network surrogates [7], as well as neural operators such as the Fourier Neural Operator (FNO) [8–11], Deep Operator Network (DeepONet) [12, 13], and In-Context Operator (ICON) [14], among others [15, 16]; unlike GPs, these are deterministic approximators and require additional methods such as ensembles or Bayesian networks for surrogate uncertainty quantification. A shared limitation of all offline surrogates is that accuracy degrades when queried out-of-distribution, which can compromise posterior fidelity [17, 18]; this can be mitigated by iterating between sampling and online surrogate refinement [2, 7], at the cost of continued training overhead.

Reviewer's specific question 2: ii) page 5. Calling "$u(.,\theta)$" a local operator is highly nonstandard as this is a function. Can this terminology be motivated properly, or revised. Is this meant to represent the map $\theta \mapsto u$ taking parameters to PDE solutions on Ω? If so please make this clear.

Response: We are glad to clarify this terminology, and respond to each part of the reviewer's concern in turn.

What is $u(\cdot,\theta)$? The function $u(\mathbf{x},\theta;W)$ takes the PDE parameter θ as an explicit input alongside the spatial coordinate $\mathbf{x}$, and its output is a field over Ω. For fixed weights W, the map $\theta \mapsto u(\cdot,\theta;W)$ sends each parameter value to a function of $\mathbf{x}$; it approximates the parameter-to-solution map $\mathcal{G} : \theta \mapsto u_\theta$ defined by $\mathcal{F}[\mathcal{G}(\theta),\theta] = 0$. We have made this precise in the revised SM, denoting the neural network approximation as $\mathcal{G}_W(\theta) = u(\cdot,\theta;W)$.

Why "local operator"? The term "operator" follows the same convention as Deep Operator Networks [12]: a network $u(\mathbf{x},\theta;W)$ that takes θ as an input and outputs a function of $\mathbf{x}$ approximates an operator from parameter space into function space. This usage is standard in the neural operator literature. The "local" qualifier distinguishes our approach from "global" operator learning. In methods such as DeepONet or FNO, the network is trained to approximate the solution for all θ in a training distribution—that is, it solves the PDE "globally" across parameter space. In B-BiLO, Conditions 1 and 2 are enforced only at the current iterate θ_k, a strictly local condition in parameter space. This is analogous to a Taylor expansion: a first-order approximation L of a function f at x_0 satisfies $L(x_0) = f(x_0)$ and $L'(x_0) = f'(x_0)$ and serves as a local model for f. Conditions 1 and 2 are the analogous first-order local consistency requirements for the parameter-to-solution map at θ_k.

Distinction from local surrogate methods. We also wish to clarify how this differs from local surrogate approaches such as [2], where a local approximation of the solution map is built in a neighborhood of θ_k and then *evaluated* at nearby parameter values during sampling, so the surrogate serves as an interpolant. As noted in our response to question (i), B-BiLO never evaluates the network away from θ_k; its role is solely to supply a differentiable computational graph at θ_k. Condition 2 is the essential ingredient: differentiating a network that merely satisfies the PDE at θ_k (Condition 1 alone) does not ensure the gradient reflects

the PDE's implicit constraint on how u changes with θ. Condition 2 closes this gap. The "local" in our terminology therefore refers to this single-point consistency requirement, not to the construction of a neighborhood interpolant.

We have added clarifying text to the manuscript making these distinctions explicit, and the term "local operator" is used consistently with the original BiLO paper [19] to distinguish from the global operator targeted by most operator learning works.

Reviewer's specific question 3: iii) Section 4.3 entitled theoretical analysis is far too terse and short to constitute a proper theoretical analysis of the method. I would ask if this can be re-labelled to directly reflect the content presented ("Some Theoretical Error Bounds" for example).

Response: We agree, and have renamed the section to "Some Theoretical Error Bounds" to better reflect the content. We have also significantly expanded the theoretical analysis by examining how the lower-level optimization in B-BiLO affects the key properties of the HMC leapfrog integrator.

The full proofs, including volume preservation (Proposition 1), approximate time-reversibility (Proposition 2), the one-step kernel perturbation bound (Proposition 3), and the main theorem (Theorem 4), are given in the SM (Sec. B). The SM section has been renamed "Theoretical Results" to better reflect the expanded content.

Reviewer's specific question 4: v) Contribution 2 on page 4 is not formulated as a contribution; it highlights the disadvantage of other methods. Also, BNNs only seem very loosely related to the aims of this paper, BNN produce uncertainty estimates over the solution field, whereas the proposed method centers on producing uncertainties on the parameters; there are distinct enough objective that I fail to see the purpose of laboring such comparisons when it is not a "gold-standard" method.

Response: We thank the reviewer for this critique. We have revised it to read as a positive statement: B-BiLO optimizes the network weights deterministically rather than sampling them, so only the low-dimensional parameter space θ is explored by the Markov chain, leading to more efficient and stable sampling.

We also agree with the reviewer that there is an important distinction between B-BiLO and BPINNs, and we appreciate the opportunity to clarify it. We note, however, that the distinction is not that B-BiLO produces uncertainty only on θ while BPINNs produce uncertainty only on u: both methods can produce uncertainty estimates on both u and θ. The real distinction lies in the *source* of uncertainty in u.

In B-BiLO, all uncertainty in u arises solely from uncertainty in θ, propagated through the PDE constraint. This is the natural and physically consistent model when the PDE is trusted: uncertainty in the solution reflects uncertainty in the model parameters. In BPINNs, uncertainty in u arises from *both* uncertainty in θ and the prior placed on the network weights W. This conflation is problematic for parameter inference, since the weight prior has no physical basis in a trusted-PDE setting, and the spurious weight uncertainty

also contaminates the marginal posterior over θ. Example 1 in the paper illustrates this directly: the PDE's symmetry guarantees $u(0) = 0$ for all k, yet BPINN produces nonzero posterior variance at the origin because individual network samples do not satisfy the PDE exactly.

If one does *not* trust the PDE model—for instance, when the PDE is only an approximation and one wishes to account for model-form error—then the stochasticity of W in BPINN can be interpreted as a form of model uncertainty. In that setting, BPINNs provide a pragmatic soft-constraint framework analogous to a misspecified model prior. Our work, however, is explicitly set in the regime where the PDE is trusted, and in that regime we argue that B-BiLO provides a more principled and efficient approach.

We retain the BPINN comparison not as a gold-standard baseline but as a methodological illustration, demonstrating the concrete consequences of treating W probabilistically when the PDE model is trusted. This comparison is relevant to practitioners who are working in the neural-solver Bayesian inference setting and may reach for BPINNs without appreciating this issue. For a more direct comparison with surrogate-based Bayesian methods—which is the approach the reviewer rightly identifies as more relevant—we have added a comparison with a pretrained DeepONet surrogate in both in-distribution and out-of-distribution settings (SM Sec. E.1, Fig. E3, Table E1). This comparison demonstrates the benefit of B-BiLO's concurrent local training over offline surrogate construction.

Reviewer's specific question 5: iv) Overall the paper lacks clarity and rigor of exposition, the notation is not idiomatic and in many parts unclear.

Response: We have carefully revised the notation and exposition throughout the manuscript, adopting a two-level convention designed to reach a broad audience while retaining mathematical precision where needed:

Main text: functions of vectors. For accessibility, the main text expresses all quantities as functions of finite-dimensional vectors. Abstract functional or operator-mapping notation are avoided. In particular:

- The PDE is written in the explicit form $F(\mathcal{D}^k u(\mathbf{x},\theta), \ldots, \mathcal{D}u(\mathbf{x},\theta), u(\mathbf{x},\theta), \theta) = 0$ for all $\mathbf{x}$.
- The neural network solution is written as $u(\mathbf{x}, \theta; W)$, where the semicolon separates the two distinct roles: θ is the physical PDE parameter of interest, and W is the neural network weight used for parametrization. This convention is used consistently for the potential energy $U(\theta; W)$ and its gradient $\nabla_\theta U(\theta; W)$.
- The likelihood is $P(D_u|\theta)$, the data misfit is $\Phi(\theta) = -\log P(D_u|\theta)$, and the potential energy is $U(\theta) = \Phi(\theta) - \log P(\theta)$.
- The residual loss L_{res} and residual-gradient loss L_{rgrad} are defined explicitly as MSE sums over collocation points.

Supplementary material (proofs): functionals and operators. In the SM, where the proofs require it, we use the standard functional analysis notation. The PDE operator is written as $\mathcal{F}[u, \theta]$, the data-misfit functional as $\Phi[\cdot]$, and the parameter-to-solution map as $\mathcal{G} : \theta \mapsto u(\cdot, \theta)$.

Convention for $(\cdot)$ vs. $[\cdot]$. Parentheses $(\cdot)$ are used for functions of vectors (e.g., $U(\theta; W)$, $u(\mathbf{x}, \theta)$), and brackets $[\cdot]$ are reserved for functionals or operators acting on function-valued arguments (e.g., $\mathcal{F}[u, \theta]$, $\Phi[u]$). The main text uses parentheses almost exclusively; brackets appear only in the SM proofs.

Notation borrowed from CES. We have partially adopted the CES notation where it improves clarity. In particular, we use $\Phi(\theta) = -\log P(D_u|\theta)$ for the data-misfit and write the potential energy explicitly as $U(\theta) = \Phi(\theta) - \log P(\theta)$, which mirrors CES's decomposition and makes the likelihood and prior contributions separately visible. We also sharpen the distinction between the exact parameter-to-solution map $\mathcal{G} : \theta \mapsto u_\theta$ and its neural-network approximation $\mathcal{G}_W(\theta) = u(\cdot, \theta; W)$ in the SM, analogous to CES's $\mathcal{G}$ vs. $\mathcal{G}^{(M)}$.

For the density notation, we retain $P(\cdot)$ throughout the main text. While $\pi(\cdot)$ is the convention used in CES and in much of the Bayesian statistics literature, $P(\cdot)$ is equally established in the inverse-problems and machine-learning literatures and is the notation used consistently in the original manuscript. We therefore keep $P(\cdot)$ to avoid introducing a notation change that would not improve precision.

Reviewer's specific question 6: page 3: "Additionally, variants of Ensemble Kalman Inversion (EnKI) can efficiently estimate the first two moments in Bayesian settings". Does EnKI recover the two first moments? I believe it only recovers a point estimate.

Can you explain the use of [.] vs (.) it seems quite inconsistently used.

Response: We thank the reviewer for this helpful comment. The original statement was imprecise. Standard Ensemble Kalman Inversion (EnKI) is primarily used to obtain a point estimate, rather than to characterize the full posterior distribution. In Bayesian variants of Kalman inversion, the ensemble mean and spread can be used to approximate the posterior mean and covariance, but this is generally justified only in the linear-Gaussian setting, or more broadly when the posterior is well approximated by a Gaussian distribution. We have revised the sentence accordingly to avoid overstating what these methods recover.

We made the following change: ... variants of **Ensemble Kalman Inversion** (EnKI) can efficiently estimate the first two moments when a Gaussian approximation is appropriate.

We thank the reviewer for pointing out the inconsistency in the notation. In the revised manuscript, we have adopted a consistent convention as described in our response to question (iv) above. Brackets $[\cdot]$ are used only for functional arguments, and parentheses $(\cdot)$ for standard function arguments.

References

[1] Cleary, E., Garbuno-Inigo, A., Lan, S., Schneider, T., Stuart, A.M.: Calibrate, emulate, sample. Journal of Computational Physics **424**, 109716 (2021) https://doi.org/10.1016/j.jcp.2020.109716

[2] Yan, L., Zhou, T.: Stein variational gradient descent with local approximations. Computer Methods in Applied Mechanics and Engineering **386**, 114087 (2021) https://doi.org/10.1016/j.cma.2021.114087

[3] Kovachki, N., Li, Z., Liu, B., Azizzadenesheli, K., Bhattacharya, K., Stuart, A., Anandkumar, A.: Neural Operator: Learning Maps Between Function Spaces. arXiv (2022). https://doi.org/10.48550/arXiv.2108.08481

[4] Pathak, J., Subramanian, S., Harrington, P., Raja, S., Chattopadhyay, A., Mardani, M., Kurth, T., Hall, D., Li, Z., Azizzadenesheli, K., Hassanzadeh, P., Kashinath, K., Anandkumar, A.: FourCastNet: A Global Data-driven High-resolution Weather Model Using Adaptive Fourier Neural Operators. arXiv (2022). https://doi.org/10.48550/arXiv.2202.11214

[5] Kennedy, M.C., O'Hagan, A.: Bayesian Calibration of Computer Models. Journal of the Royal Statistical Society Series B: Statistical Methodology **63**(3), 425–464 (2001) https://doi.org/10.1111/1467-9868.00294

[6] Lan, S., Li, S., Shahbaba, B.: Scaling Up Bayesian Uncertainty Quantification for Inverse Problems Using Deep Neural Networks. SIAM/ASA Journal on Uncertainty Quantification **10**(4), 1684–1713 (2022) https://doi.org/10.1137/21M1439456

[7] Li, Y., Wang, Y., Yan, L.: Surrogate modeling for Bayesian inverse problems based on physics-informed neural networks. Journal of Computational Physics **475**, 111841 (2023) https://doi.org/10.1016/j.jcp.2022.111841

[8] Li, Z., Kovachki, N., Azizzadenesheli, K., Liu, B., Bhattacharya, K., Stuart, A., Anandkumar, A.: Fourier Neural Operator for Parametric Partial Differential Equations. arXiv (2021). https://doi.org/10.48550/arXiv.2010.08895

[9] Li, Z., Zheng, H., Kovachki, N., Jin, D., Chen, H., Liu, B., Azizzadenesheli, K., Anandkumar, A.: Physics-Informed Neural Operator for Learning Partial Differential Equations. ACM / IMS Journal of Data Science **1**(3), 9–1927 (2024) https://doi.org/10.1145/3648506

[10] White, C., Berner, J., Kossaifi, J., Elleithy, M., Pitt, D., Leibovici, D., Li, Z., Azizzadenesheli, K., Anandkumar, A.: Physics-Informed Neural Operators with Exact Differentiation on Arbitrary Geometries. In: The Symbiosis of Deep Learning and Differential Equations III (2023)

[11] Akyildiz, O.D., Girolami, M., Stuart, A.M., Vadeboncoeur, A.: Efficient Prior Calibration from Indirect Data. SIAM Journal on Scientific Computing **47**(4), 932–958 (2025) https://doi.org/10.1137/24M166485X

[12] Lu, L., Jin, P., Pang, G., Zhang, Z., Karniadakis, G.E.: Learning nonlinear operators via DeepONet based on the universal approximation theorem of operators. Nature Machine Intelligence **3**(3), 218–229 (2021) https://doi.org/10.1038/s42256-021-00302-5

[13] Wang, S., Wang, H., Perdikaris, P.: Learning the solution operator of parametric partial differential equations with physics-informed DeepONets. Science Advances **7**(40), 8605 (2021) https://doi.org/10.1126/sciadv.abi8605

[14] Yang, L., Liu, S., Meng, T., Osher, S.J.: In-context operator learning with data prompts for differential equation problems. Proceedings of the National Academy of Sciences **120**(39), 2310142120 (2023) https://doi.org/10.1073/pnas.2310142120

[15] O'Leary-Roseberry, T., Chen, P., Villa, U., Ghattas, O.: Derivative-Informed Neural Operator: An efficient framework for high-dimensional parametric derivative learning. Journal of Computational Physics **496**, 112555 (2024) https://doi.org/10.1016/j.jcp.2023.112555

[16] Molinaro, R., Yang, Y., Engquist, B., Mishra, S.: Neural Inverse Operators for Solving PDE Inverse Problems. arXiv (2023). https://doi.org/10.48550/arXiv.2301.11167

[17] Nelsen, N.H., Yang, Y.: Operator Learning Meets Inverse Problems: A Probabilistic Perspective. arXiv (2025). https://doi.org/10.48550/arXiv.2508.20207

[18] Cao, L., O'Leary-Roseberry, T., Jha, P.K., Oden, J.T., Ghattas, O.: Residual-based error correction for neural operator accelerated infinite-dimensional Bayesian inverse problems. Journal of Computational Physics **486**, 112104 (2023) https://doi.org/10.1016/j.jcp.2023.112104

[19] Zhang, R.Z., Miles, C.E., Xie, X., Lowengrub, J.S.: BiLO: Bilevel Local Operator Learning for PDE Inverse Problems. Journal of Computational Physics **551**, 114679 (2026) https://doi.org/10.1016/j.jcp.2026.114679

Response to Reviewers

We thank all reviewers for their time and constructive feedback throughout the review process. In this document, comments from the reviewers are shown in blue, our responses are in black, and revisions to the manuscript are quoted in red.

Reviewer #1

Reviewer's comment: I thank the authors for their careful and substantial revision of the manuscript. The paper has improved considerably since the previous version. Several of my earlier concerns have been addressed satisfactorily, and I no longer see these as obstacles to publication.

Response: We thank the reviewer for their careful reading of our manuscript across the revision rounds, and we are pleased that our revisions have addressed their concerns.

Reviewer's comment: (Remarks on code availability) The authors have provided code that appears to be a useful companion to the paper. The implementation is reasonably well-organized and well-documented, and the available instructions seem sufficient for a reader to install the required dependencies and run the main examples.

I inspected the code but did not independently verify every experiment. Based on my review, the code appears to support the reproducibility of the manuscript's main numerical results and should be a useful resource for the community.

Response: We thank the reviewer for taking the time to inspect our code and for the positive assessment of its organization, documentation, and reproducibility.

Reviewer #2

Reviewer's comment: I co-reviewed this manuscript with one of the reviewers who provided the listed reports. This is part of the Nature Communications initiative to facilitate training in peer review and to provide appropriate recognition for Early Career Researchers who co-review manuscripts.

Response: We thank the reviewer for their contribution to the review of our manuscript.

Reviewer #3

Reviewer's comment: The revised draft addresses most of my concerns, except for my final comment regarding the potential limitations in high-dimensional settings. The issue is not merely computational cost. In high-dimensional problems, HMC can be difficult to tune

and may suffer from low acceptance rates, wasted computation, or divergent transitions. In this sense, if the dimension of the inferred known parameter space becomes large, the proposed method may face substantial practical challenges. I encourage the authors to discuss this limitation and its potential impact on the applicability of their approach.

Response: We thank the reviewer for this important point, and we agree. Our previous response focused on the computational cost per effective sample, whereas the reviewer's concern is about the behavior of the sampler itself in high dimensions, which is a distinct issue. Since the upper level of B-BiLO is standard HMC, the framework inherits some known challenges of HMC in high-dimensional settings: sensitivity to the tuning of the step size, trajectory length, and mass matrix, and the possibility of low acceptance rates, wasted computation, or divergent transitions. Indeed, our scalability study already exhibits one aspect of this issue: with fixed leapfrog settings, the per-trajectory displacement of θ grows as $\sqrt{K}$, which drives up the lower-level fine-tuning cost and, in substantially higher dimensions, would eventually require retuning the step size to maintain a reasonable acceptance rate. Moreover, these challenges are amplified in B-BiLO, because each leapfrog step invokes lower-level fine-tuning, so rejected or wasted trajectories are more costly than in classical HMC.

We have added an explicit discussion of this limitation, and of the remedies that are compatible with our framework, to the Discussion section of the revised manuscript:

In the current implementation, the upper level of B-BiLO is standard HMC, and thus the framework inherits the known challenges of HMC in high-dimensional settings: performance is sensitive to the tuning of the step size, trajectory length, and mass matrix, and maintaining a reasonable acceptance rate requires decreasing the step size as the dimension grows; poorly tuned samplers can suffer from low acceptance rates, wasted computation, or divergent transitions [1, 2]. These challenges are amplified in B-BiLO, where each leapfrog step invokes lower-level fine-tuning of a local operator, so rejected trajectories are more costly than in classical HMC. Because the upper level requires only gradient evaluations of the potential energy, B-BiLO is compatible with standard remedies, including adaptive step-size and mass-matrix tuning [2] and, for unknown-function inference with Gaussian priors, dimension-robust function-space samplers [3]; integrating such samplers is a promising direction for future work.

References

[1] Neal, R.M.: MCMC Using Hamiltonian Dynamics. In: Handbook of Markov Chain Monte Carlo. Chapman and Hall/CRC

[2] Hoffman, M.D., Gelman, A.: The No-U-Turn Sampler: Adaptively Setting Path Lengths in Hamiltonian Monte Carlo. Journal of Machine Learning Research **15**(47), 1593–1623 (2014)

[3] Beskos, A., Girolami, M., Lan, S., Farrell, P.E., Stuart, A.M.: Geometric MCMC for infinite-dimensional inverse problems. Journal of Computational Physics **335**, 327–351 (2017) `https://doi.org/10.1016/j.jcp.2016.12.041`
